# Exploratory study of non-ordinary states of consciousness during sleep show distinct electrophysiological features from wakefulness and canonical sleep stages

**DOI:** 10.1038/s41598-025-18748-7

**Published:** 2025-09-29

**Authors:** Nerea L. Herrero, Yohann Corfdir, Aylin A. Vázquez-Chenlo, Lucila Capurro, Cecilia Forcato

**Affiliations:** Laboratorio de Sueño y Memoria, Departamento de Ciencias de La Vida, Instituto Tecnológico de Buenos Aires (ITBA), Iguazú 341, (1437) Capital Federal, Buenos Aires, Argentina

**Keywords:** Neuroscience, Cognitive neuroscience

## Abstract

Consciousness does not always fade during sleep. Instead, it can re-emerge in the form of non-ordinary states of consciousness (NOSC), such as lucid dreams (LDs), sleep paralysis (SP), out-of-body experiences (OBEs), and false awakenings (FAs). While some of these states have been studied phenomenologically, their neurophysiological underpinnings remain unclear. In this exploratory study, we investigate their electrophysiological correlates and distinguish them from standard sleep stages. We conducted overnight polysomnography in frequent experiencers, capturing 10 episodes (3 LDs, 2 SP, 2 OBEs, 3 FAs). Eye movement markers identified periods of lucidity. Relative spectral power was analyzed using principal component analysis (PCA) and permutation-based multivariate analysis of variance (PERMANOVA). Our results indicate that these NOSC are distinct from wakefulness, yet share features with both stage 1 (S1) and rapid eye movement (REM) sleep.

**Supplementary Information:**

The online version contains supplementary material available at 10.1038/s41598-025-18748-7.

## Introduction

During wakefulness, all our experiences, thoughts, emotions, and feelings combine and unfold as a whole, resulting in the unique and irreplaceable sensation of having a subjective experience and thus being individuals endowed with consciousness. Consciousness remains stable during wakefulness, and while it may be absent during certain moments of sleep, it can also emerge in specific non-ordinary states of consciousness such as lucid dreams (LDs), sleep paralysis (SP), out-of-body experiences (OBEs) and false awakenings (FAs). In this study, we refer to these phenomena as non-ordinary states of consciousness during sleep (NOSC), a term we adopt to describe conscious states that differ from both ordinary wakefulness and typical non-lucid dreaming. These states involve distinctive experiential features and the preservation of reflective awareness. This approach aligns with broader efforts to develop inclusive taxonomies of conscious states beyond ordinary waking experience^1^.

LDs are dreams in which individuals become aware that they are dreaming. During a LD, people can act voluntarily within the dream and are often able to recall and execute pre-planned actions that were agreed upon before sleep^2–4^. LDs are primarily a REM sleep phenomenon, with few exceptions^5^. Lucid dreamers can signal the onset of their lucid state through pre-arranged eye movements, enabling communication with researchers in laboratory settings. Electroencephalographic (EEG) studies have identified distinct brain activity associated with lucid dreaming, such as an increase in low-beta frequencies (13–19 Hz) in parietal regions^3^ and an increase in frontolateral low-gamma activity (40 Hz)^6,7^. However, recent studies have questioned the previously reported low-gamma increase during this state. For instance, Baird et al. (2022) argued that this phenomenon might be an artifact caused by saccadic spike potentials, which are linked to the heightened REM density observed during LDs^8^. Conversely, a more recent study reported augmented low-gamma activity during LDs, leaving the role of low-gamma inconclusive^9^.

SP is a parasomnia characterized by the temporary inability to perform voluntary movements during the transitions between sleep and wakefulness^10,11^. During an SP episode, individuals often perceive themselves as being awake and aware of their surroundings, despite still being in a sleep state. SP can cause significant distress due to the presence of negative emotions and vivid hallucinations that commonly accompany episodes^12,13^. EEG studies on episodes of SP are scarce and have been conducted in both healthy individuals^14–16^ and patients with narcolepsy^14,17,18^. Most of these studies performed qualitative analyses of the EEG signal, highlighting the presence of “alpha trains” during SP^15–17^. More recent findings, however, have revealed a significant increase in relative alpha power during SP^14^ accompanied by a significant decrease in relative theta power compared to REM sleep (Mainieri et al., 2021). Additionally, Terzaghi et al. (2012) described the coexistence of alpha-like rhythms and high-frequency peaks (~ 18 Hz) during SP^18^.

OBEs are an altered state of consciousness in which individuals perceive themselves as being outside their physical body, observing the world from an external perspective^19,20^. OBEs have been reported across various cultures throughout history and remain one of the great mysteries in the study of human consciousness. They are often described as extremely vivid, with perceptual qualities similar to veridical perception^13,19^, and are associated with lasting psychological and existential changes, including increased spirituality, reduced fear of death, heightened empathy, and an expanded perception of reality^21–23^. Neuroscientific research suggests that OBEs may result from a temporary disruption in the integration of multisensory information, with the temporo-parietal junction (TPJ) playing a critical role in this process^20,24,25^. OBEs associated with sleep are often linked to other phenomena, such as LD, SP, and FAs^13,26,27^.

FAs are dreams in which the subjects have an erroneous belief that they are waking up in a familiar place, starting a daytime routine, only to later realize that they are still dreaming^28^. There are only two EEG studies of FA published^14,16^. Of these, Takeuchi et al. (1992) did not formally distinguish FAs from other episodes and included them within the broader category of SP in their analyses. In contrast, Mainieri et al. (2021) conducted a detailed examination of FAs, finding that their EEG profile closely resembles SP, characterized by a significant reduction in theta relative power and a notable increase in alpha relative power compared to REM sleep^14^.

Here, we aimed to characterize the electrophysiological correlates of different NOSC during sleep and to differentiate these states from standard sleep stages, such as REM sleep and Stage 1 sleep (S1), as well as wakefulness. S1 is formally categorized as the lightest stage of non-REM sleep, yet it is also characterized by transitional electrophysiological features and the frequent occurrence of hypnagogic experiences^29^. These attributes make S1 a meaningful comparison condition when exploring NOSC which often emerge at the boundaries between wakefulness and sleep. Because experiences such as lucid dreaming and sleep paralysis have been conceptualized as hybrid states, sharing features of both wakefulness and sleep^7,10,14^, we considered S1 a particularly relevant point of comparison, alongside REM sleep and wakefulness.

## Results

**Fig. 1:**
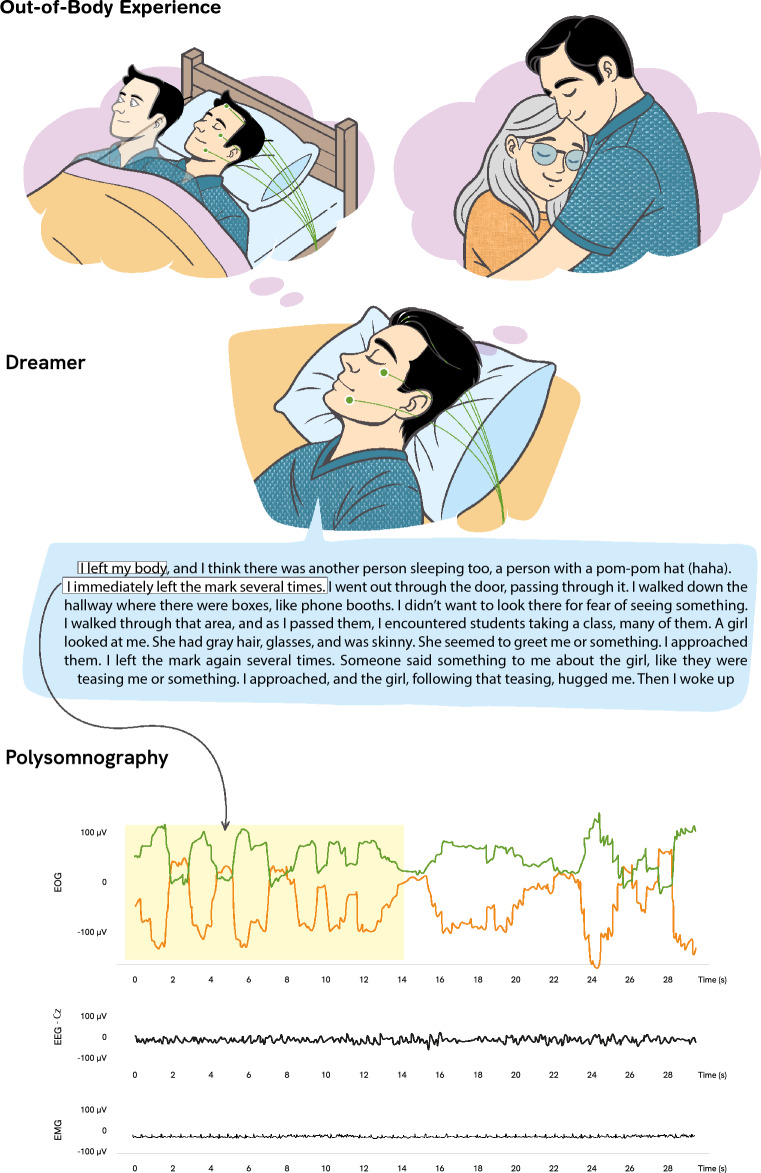
Representation of one of the NOSC of the study (OBE_1_). The figure presents the participant’s report describing the experience, alongside the corresponding polysomnographic recording, showing real-time physiological activity and pre-agreed eye movement signals. The PSG data were extracted from original recordings collected during the study. The illustrations were created by the first author (NLH), using the AI platform Recraft as a base, and subsequently edited and finalized in Adobe Illustrator (Version 25.0.1, 2021, https://www.adobe.com/products/illustrator.html).

To characterize LDs, SP, OBEs, and FAs, we conducted full-night polysomnographic recordings in the laboratory with individuals who frequently experience these states (n = 7; 3 LDs, 2 SP, 2 OBEs, 3 FAs). Participants were trained to perform a predefined eye movement signal upon becoming aware during sleep, allowing us to identify the onset of NOSC (Fig. 1). EEG data were preprocessed to remove ocular artifacts using independent component analysis (ICA), and power spectral density (PSD) was computed for each segment of interest. Thus, first to investigate spectral patterns associated with each NOSC, we applied principal component analysis (PCA), a dimensionality reduction technique that linearly transforms the original correlated spectral variables into a new set of orthogonal, uncorrelated components, ranked by the amount of variance they capture from the data. This transformation preserves the structure of the data while allowing for a more compact and interpretable representation in a lower-dimensional space. Second, we conducted permutation-based multivariate analyses of variance (PERMANOVA) on an individual-subject basis to statistically assess differences in EEG spectral activity across states, followed by post-hoc analyses.

### Lucid dreaming

**Fig. 2:**
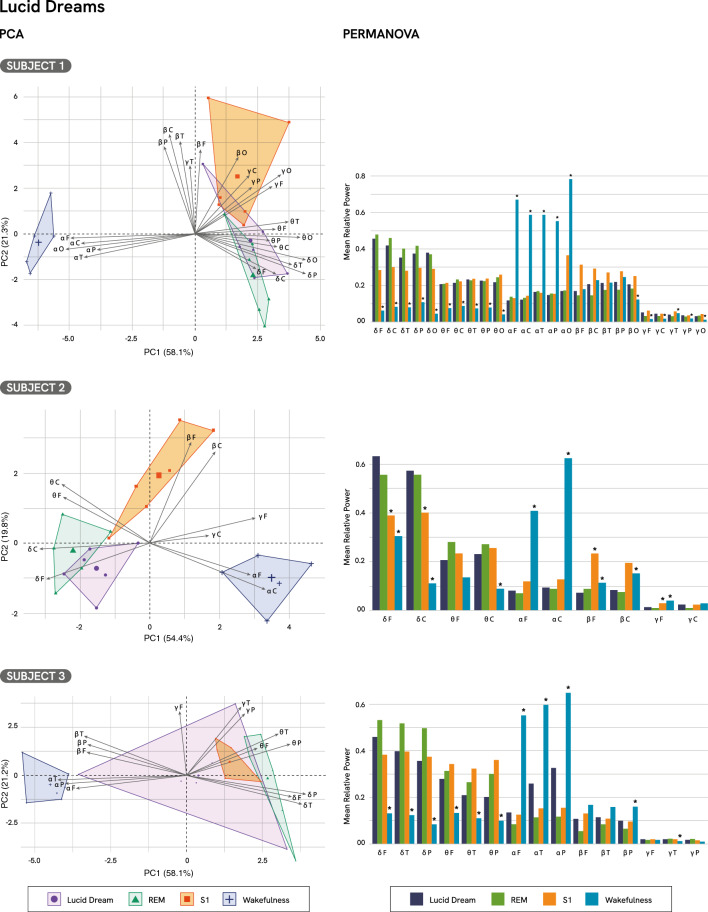
PCA and PERMANOVA results for lucid dreams (LD) in Subjects 1, 2, and 3. PCA biplots (left) show the distribution of LD, REM sleep, stage 1 sleep (S1), and wakefulness across the first two principal components, which together explain the majority of spectral variance. Contribution and Cos^2^ of Subject 1, LD: 24%, 0.590, respectively; REM sleep 40%, 0.636; S1 57%, 0.494; wakefulness 78%, 0.957. Subject 2, LD: 24%, 0.561; REM sleep 31%, 0.728; S1 total 69%, 0.767; wakefulness 75%, 0.759 and Subject 3, LD: 51%, 0.454; REM sleep 64%, 0.803; S1 16%, 0.538; wakefulness 67%, 0.921. PERMANOVA results revealed significant differences in mean relative spectral power between conditions. Post hoc comparisons were performed between LD and each of the standard sleep stages (REM sleep, S1, and wakefulness). Asterisks (*) indicate statistically significant pairwise differences (p < 0.05).

PCA showed that the first two components, explaining between 73 and 79% of the total variance across subjects, reliably represented the topographical organization of LD, REM sleep, S1, and wakefulness (Fig. 2). In all three subjects, LD episodes were clearly separated from wakefulness in the PCA space, showing greater association with delta and theta activity independently of the region. Importantly, LDs showed partial overlap with REM sleep and S1 in the PCA space, but with subject-specific patterns. In Subjects 1 and 3, LDs were also associated with minor contributions from the beta and low-gamma bands, while in Subject 3, one segment of the LD episode additionally exhibited contributions from the alpha band. For a comprehensive analysis, see Supplementary Tables S1–S3, and Supplementary Figures S1–S3.

Additionally, PERMANOVA revealed significant differences in oscillatory activity across the states of consciousness (LDs, REM sleep, S1 and wakefulness) (Fig. 2; Subject_1_: F(3,20) = 36.499, p < 0.001, R^2^ = 0.845; Subject_2_: F(3,20) = 24.705, p < 0.001, R^2^ = 0.788; Subject_3_: F(3,20) = 15.831, p < 0.001, R^**2**^ = 0.704). Specifically, post-hoc analysis revealed no significant differences between LDs and REM sleep (Fig. 2; PERMANOVA, Subject_1_: F(1,10) = 0.563, p = 1.000, R^2^ = 0.054; Subject_2_: F(1,10) = 1.597, R^2^ = 0.140, p = 1.000; Subject_3_: F(1,10) = 2.534, p = 0.522, R^2^ = 0.202; Supplementary Tables S4, S6, and S9). Differences between LDs and S1 were variable across subjects. In Subject 2, LD significantly differed from S1 (PERMANOVA, Subject_2_: F(1,10) = 12.345, R^2^ = 0.552, p = 0.017), characterized by increased delta and reductions in beta relative power in fronto-central regions, as well as decreased frontal low-gamma activity (Table 1; Supplementary Table S7). Subjects 1 and 3 showed no significant differences compared to S1, consistent with their overlap in PCA space (PERMANOVA, Subject_1_: F(1,10) = 3.244, p = 0.217, R^2^ = 0.245; Subject_3_: F(1,10) = 2.303, p = 0.500, R^2^ = 0.187). Interestingly, all LDs differed significantly from wakefulness (PERMANOVA, Subject_1_: F(1,10) = 72.452, p = 0.011, R^2^ = 0.879, Subject_2_: F(1,10) = 46.144, p = 0.013, R^2^ = 0.822, Subject_3_: F(1,10) = 15.275, p = 0.013, R^2^ = 0.604). Post-hoc PERMANOVA results revealed that differences between these two states were driven by increases in delta and theta relative power, and decreases in alpha, consistently across all regions, except for Subject 2 where the increase in theta was only in the central region (Supplementary Tables S5, S8 and S10). For beta relative power, Subject 2 showed decreases across frontal regions, while Subject 3 exhibited a decrease limited to the parietal region. In contrast, Subject 1 showed increased beta relative power in the occipital area. Regarding low-gamma, Subject 1 displayed a decrease in the temporal region along with increases in frontal, central, parietal, and occipital areas. Subject 2 showed reduced frontal low-gamma activity, while Subject 3 exhibited increased low-gamma relative power in the temporal region.

**Table 1:** Spectral power differences between lucid dreaming and standard sleep stages (PERMANOVA results).

Subject	Sleep Stage	Delta	Theta	Alpha	Beta	Low-Gamma
1	REM	**ns**	**ns**	**ns**	**ns**	**ns**
2	REM	**ns**	**ns**	**ns**	**ns**	**ns**
3	REM	**ns**	**ns**	**ns**	**ns**	**ns**
1	S1	ns	**ns**	**ns**	ns	ns
2	S1	↑ frontal, central	**ns**	**ns**	↓ frontal, central	↓ frontal
3	S1	ns	**ns**	**ns**	ns	ns
1	Wakefulness	↑ **all regions**	↑ **all regions**	↓ **all regions**	↑ occipital	↑ frontal,central, parietal,occipital ↓ temporal
2	Wakefulness	↑ **all regions**	↑ **central**	↓ **all regions**	↓ frontal	↓ frontal
3	Wakefulness	↑ **all regions**	↑ **all regions**	↓ **all regions**	↓ parietal	↑ temporal

Summary of significant spectral power differences (PERMANOVA) between episodes of lucid dreaming (LD) and three Standard Sleep Stages: REM sleep, Stage 1 sleep (S1), and wakefulness. Arrows indicate significant increases (↑) or decreases (↓) in relative power for each frequency band, with scalp regions specified. “ns” denotes non-significant comparisons. Common results across subjects are shown in bold.

### Sleep paralysis

**Fig. 3:**
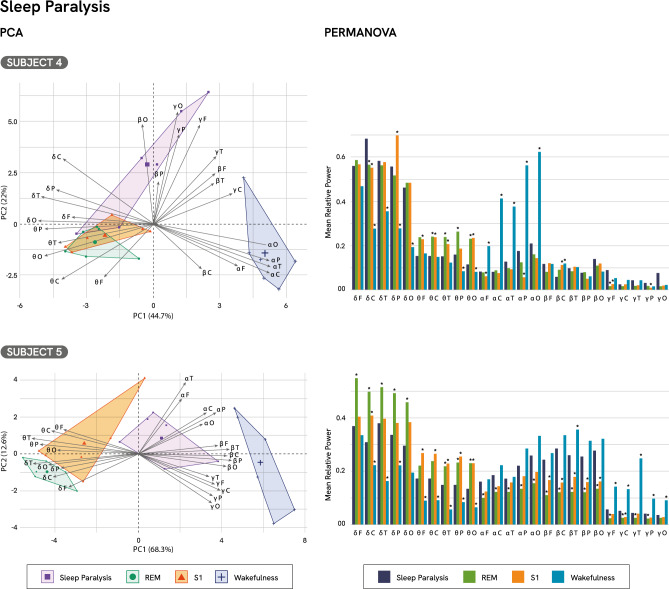
PCA and PERMANOVA results for sleep paralysis.

PCA results showed that the first two principal components explained between 66 and 80% of the variance across subjects (Fig. 3). In Subject 4, the SP episode was positioned in a region of the PCA space associated with beta and low-gamma activity. In Subject 5, SP exhibited contributions from the alpha and beta bands. In both cases, SP showed reduced influence from delta and theta activity compared to REM sleep. Moreover, SP episodes formed a distinct cluster in the PCA space, separate from REM sleep, S1, and wakefulness, highlighting the distinct neural profile of this NOSC. For a comprehensive analysis, see Supplementary Tables S11 and S12, and Supplementary Figures S4 and S5.

PERMANOVA revealed robust differences in spectral activity across states of consciousness for both subjects (Fig. 3, PERMANOVA, Subject_4_: F(3,20) = 18.832, p < 0.001, R^**2**^ = 0.738, Subject_5_: F(3,20) = 17.174, p < 0.001, R^**2**^ = 0.720). Compared to REM, both episodes showed significant differences (PERMANOVA, Subject_4_: F(1,10) = 5.367, p = 0.014, R^2^ = 0.361; Subject_5_: F(1,10) = 17.87, p = 0.016, R^2^ = 0.64; Supplementary Tables S13 and S17). In particular, both episodes were characterized by notable decreases in theta relative power (Table 2; Supplementary Tables S14 and S18). While both subjects showed reductions in temporal, parietal, and occipital regions, subject 4 also exhibited decreased relative power in central areas. Delta activity yielded mixed results, as Subject 4 exhibited a significant increase in the central region, while Subject 5 showed reductions across all regions, rendering the role of delta activity inconclusive. Both episodes showed increased low-gamma relative power. While both subjects exhibited increases in frontal regions, Subject 5 also showed heightened relative power in central, temporal, and parietal areas. Subject 5 also demonstrated significant increases in alpha and beta activity across all regions.

**Table 2:** Spectral power differences between sleep paralysis and standard sleep stages (PERMANOVA Results).

Subject	Sleep Stage	Delta	Theta	Alpha	Beta	Low-Gamma
4	REM	↑ central	↓ **central, temporal, parietal, occipital**	ns	ns	↑ **frontal**
5	REM	↓ all regions	↓ **temporal, parietal, occipital**	↑ all regions	↑ all regions	↑ **frontal, central, temporal, parietal**
4	S1	↓ parietal ↑ central	↓ **frontal, central, temporal, occipital**	↑ frontal, parietal	↓ central	↑ **frontal, parietal**
5	S1	↓ central	↓ **all regions**	ns	↑ all regions	↑ **central**
4	Wakefulness	↑ **central, temporal, parietal, occipital**	↑ **parietal, occipital**	↓ all regions	↓ **central**	ns
5	Wakefulness	↑ **central, temporal, parietal**	↑ **all regions**	ns	↓ **temporal**	↓ all regions

Summary of significant spectral power differences (PERMANOVA) between episodes of sleep paralysis (SP) and three Standard Sleep Stages: REM sleep, Stage 1 sleep (S1), and wakefulness. Arrows indicate significant increases (↑) or decreases (↓) in relative power for each frequency band, with scalp regions specified. “ns” denotes non-significant comparisons. Common results across subjects are shown in bold.

Compared to S1, both SP exhibited significant differences (PERMANOVA, Subject_4_: F(1,10) = 5.462, p = 0.042, R^2^ = 0.353; Subject_5_: F(1,10) = 5.895, p = 0.031, R^2^ = 0.370). Specifically, theta relative power was significantly reduced in both episodes (Supplementary Tables S15 and S19). Subject 4 exhibited reductions in the frontal, central, temporal, and occipital regions and Subject 5 showed decreases across all regions. Regarding delta activity, Subject 4 exhibited a significant decrease in parietal delta accompanied by an increase in central delta. Conversely, Subject 5 exhibited a significant decrease in central delta, further highlighting the inconclusive role of delta activity in SP. Furthermore, both SP episodes showed a significant increase in low-gamma activity. While Subject 4 exhibited widespread increases in frontal and parietal regions, Subject 5 showed a more localized increase in central areas. Additionally, the two subjects showed contrasting patterns in beta relative power. Subject 4 displayed decreased activity in central areas, while Subject 5 exhibited widespread increases across all regions. Subject 4 also showed increased alpha activity in fronto-parietal areas, a pattern not observed in Subject 5.

Compared to wakefulness, both episodes showed significant differences (PERMANOVA, Subject_4_: F(1,10) = 21.756, p = 0.016, R^2^ = 0.685; Subject_5_: F(1,10) = 11.123, p = 0.013, R^2^ = 0.526). Both subjects showed significant increases in delta and theta relative power (Supplementary Tables S16 and S20). For delta relative power, both subjects exhibited increases in central, temporal, and parietal regions; however, only Subject 4 showed an additional increase in the occipital area. Regarding theta activity, Subject 4 displayed increased relative power in parieto-occipital regions, while Subject 5 exhibited increases across all regions. Furthermore, significant reductions in alpha and beta relative power were observed. Subject 4 showed decreased alpha relative power across all regions. Regarding beta relative power, Subject 4 exhibited a decrease in the central region, whereas Subject 5 showed a decrease in the temporal region. Additionally, Subject 5 showed a widespread reduction in low-gamma relative power across all regions.

The PCA biplots illustrate the distribution of SP, wakefulness, REM sleep, and S1 along the first two principal components, which together capture the majority of spectral variance. Contribution and Cos^2^ of Subject 4, SP: 76%, 0.561, respectively; REM sleep: 23%, 0.571; S1: 18%, 0.454; wakefulness: 81%, 0.793. Subject 5, SP: 20%, 0.423; REM sleep: 39%, 0.869; S1: 40%, 0.759; wakefulness: 98%, 0.885. PERMANOVA results revealed significant differences in mean relative spectral power between conditions. Post hoc comparisons were performed between SP and each of the standard sleep stages (REM sleep, S1, and wakefulness). Asterisks (*) indicate statistically significant pairwise differences (p < 0.05).

### Out-of-body experiences

**Fig. 4:**
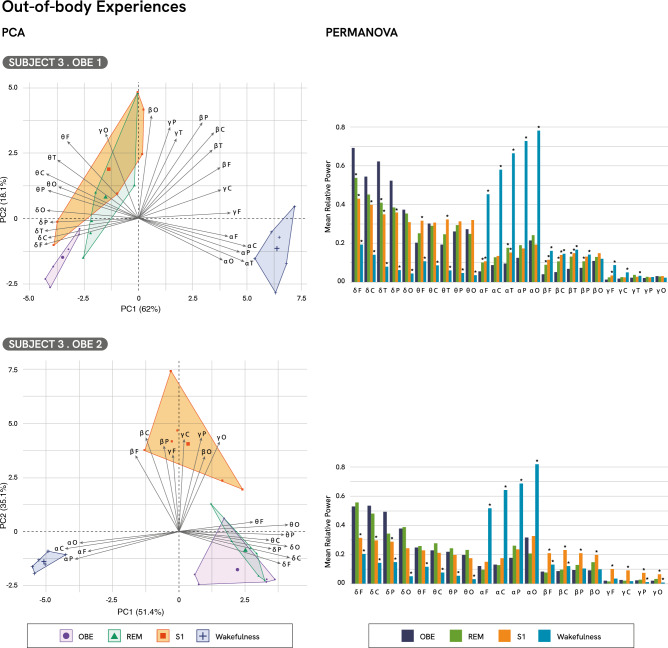
PCA and PERMANOVA results for the OBEs from Subject 3. The PCA biplot illustrates the distribution of OBEs, wakefulness, REM sleep, and S1 across the principal components. Contribution and Cos^2^ of Subject 3, OBE_1_: 36%, 0.741 respectively; REM sleep: 31%, 0.604; S1: 52%, 0.667; wakefulness: 79%, 0.879. Subject 3, OBE_2_: 31%, 0.751; REM sleep: 24%, 0.700; S1: 73%, 0.799; wakefulness: 70%, 0.957. PERMANOVA results revealed significant differences in mean relative spectral power between conditions. Post hoc comparisons were performed between OBE and each of the standard sleep stages (REM sleep, S1, and wakefulness). Asterisks (*) indicate statistically significant pairwise differences (p < 0.05).

PCA results for Subject 3 indicated that the first two principal components captured between 80 and 85% of the total variance (Fig. 4). Both OBE_1_ and OBE_2_, were strongly shaped by delta and theta activity, and occupied regions clearly separated from wakefulness and S1. OBE_1_, in particular, was more distinctly separated from REM sleep, projecting along components primarily defined by delta and theta bands, while OBE_2_ showed partial overlap with REM sleep. Full PCA details are reported in Supplementary Tables S21 and S22, and Supplementary Figures S6 and S7.

PERMANOVA confirmed a significant effect of the state of consciousness on oscillatory activity for both, OBE_1_ (F(3, 20) = 53.134, p < 0.001, R^2^ = 0.889) and OBE_2_ (F(3, 20) = 15.831, p < 0.001, R^2^ = 0.704). Specifically, we found significant differences between OBE_1_ and REM sleep (PERMANOVA, F(1,10) = 6.075, p = 0.049, R^2^ = 0.378; Supplementary Table S23), but no for OBE_2_ (PERMONOVA, F(1,10) = 1.524, p = 1.000, R^2^ = 0.132; Supplementary Table S27). OBE_1_ exhibited significant increases in delta relative power and reductions in the relative power of alpha and beta activity in widespread regions (Table 3; Supplementary Table S24).

**Table 3:** Spectral power differences between OBEs and standard sleep stages (PERMANOVA Results).

Subject	Sleep stage	Delta	Theta	Alpha	Beta	Low-Gamma
3 – OBE_1_	REM	↑ frontal, temporal	**ns**	↓ frontal, temporal	↓ frontal, central, temporal	**ns**
3 – OBE_2_	REM	ns	**ns**	ns	ns	ns
3 – OBE_1_	S1	↑ **frontal, central, temporal, parietal**	↓ fronto-temporal	↓ frontal, temporal	↓ **frontal, central, temporal**	↓ **frontal**
3 – OBE_2_	S1	↑ **frontal, central, parietal**	ns	ns	↓ **all regions**	↓ **all regions**
3 – OBE_1_	Wakefulness	↑ **all regions**	↑ **all regions**	↓ **all regions**	↓ **frontal, central, temporal, parietal**	↓ **frontal, central, temporal**
3 – OBE_2_	Wakefulness	↑ **all regions**	↑ **all regions**	↓ **all regions**	↓ **frontal, central**	↑ **parietal, occipital**

Summary of significant spectral power differences (PERMANOVA) between out-of-body experiences (OBE) and three Standard Sleep Stages: REM sleep, Stage 1 sleep (S1), and wakefulness. Arrows indicate significant increases (↑) or decreases (↓) in relative power for each frequency band, with scalp regions specified. “ns” denotes non-significant comparisons. Common results across subjects are shown in bold.

Both OBEs showed significant differences compared to S1 (PERMANOVA, OBE_1_: F(1,10) = 11.652, p = 0.029, R^2^ = 0.538; OBE_2_: F(1,10) = 11.616, p = 0.017, R^2^ = 0.537). Both were marked by increased delta relative power in frontal, central and parietal regions (Supplementary Tables S25 and S28). The main difference between them was the additional involvement of the temporal region in OBE_1_. A significant reduction in theta and alpha activity in fronto-temporal regions was observed in OBE_1_, but no significant differences were observed in OBE_2_. Beta activity decreased in frontal, central, and temporal regions during OBE_1_, whereas in OBE_2_, the decrease extended across all regions. Similarly, low-gamma activity was reduced in both OBEs, specifically in frontal regions in OBE_1_, but extended across all regions in OBE_2_.

There was a significant difference between wakefulness and both OBEs (PERMANOVA, OBE_1_ F(1,10) = 178.139, p = 0.010, R^2^ = 0.947; OBE_2_ F(1,10) = 74.655, p = 0.013, R^2^ = 0.882). Particularly, both OBEs showed significant increases in delta and theta relative power, as well as reduction in alpha activity across all regions (Supplementary Tables S26 and S29). In addition, significant decreases were observed in fronto-central beta relative power for both OBEs, but OBE_1_ also exhibited increases in temporal and parietal regions.

Finally, OBE_1_ exhibited significant reductions in low-gamma relative power across frontal, central, and temporal regions, in contrast to OBE_2_, which showed increases in parieto-occipital areas.

### False awakenings

**Fig. 5:**
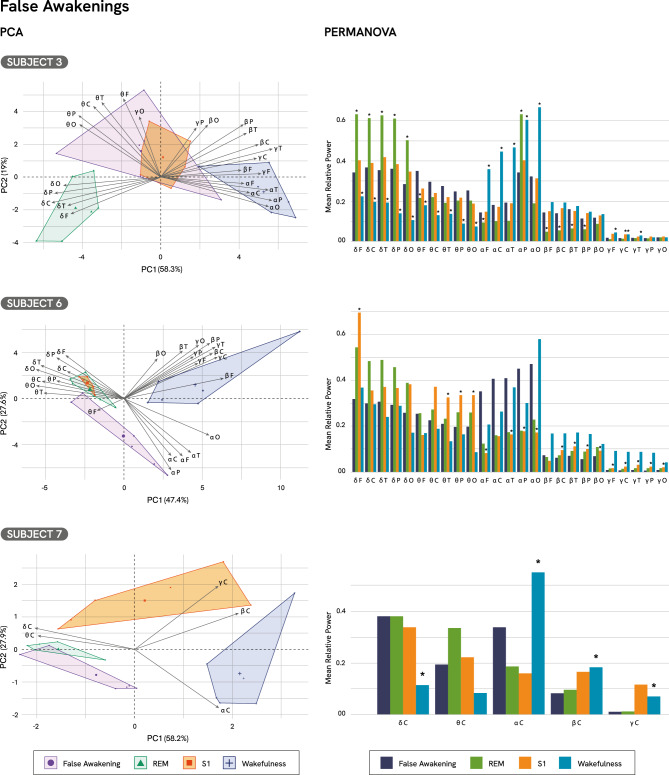
PCA and PERMANOVA results for False Awakenings from Subjects 3, 6 and 7. The PCA biplot illustrates the distribution of False Awakenings (FA), REM sleep, S1 and wakefulness across the principal components. Contribution and Cos^2^ of Subject 3, FA: 47%, 0.557; REM sleep: 68%, 0.849; S1: 19%, 0.446; wakefulness: 64%, 0.787. Subject 6, FA: 31%, 0.751; REM sleep: 24%, 0.700; S1: 73%, 0.799; wakefulness: 70%, 0.957. Subject 7, FA: 28%, 0.802; REM sleep: 24%, 0.801; S1: 68%, 0.830; wakefulness: 77%, 0.971. PERMANOVA results revealed significant differences in mean relative spectral power between conditions. Post hoc comparisons were performed between FA and each of the standard sleep stages (REM sleep, S1, and wakefulness). Asterisks (*) indicate statistically significant pairwise differences (p < 0.05).

PCA results for FA indicated that the first two principal components accounted for between 74 and 85% of the total variance across subjects (Fig. 5). The analysis revealed heterogeneous spectral profiles across the three FA episodes. Specifically, FA remained distinct from wakefulness in the PCA space for all three subjects. In Subject 3, the FA episode was characterized by strong expression of beta and low-gamma activity, alongside theta, and occupied a distinct position in PCA space, clearly separated also from REM sleep and S1. In contrast, Subject 6’s FA was dominated by alpha activity, with moderate contributions from beta, low-gamma, theta, and delta, clustering separately from both REM sleep and S1. Subject 7 exhibited a mixed spectral profile characterized by alpha, theta, and delta activity. This episode was clearly distinct from S1, while partially overlapping with REM sleep. Full PCA results, including contributions and loadings, are reported in Supplementary Tables S30–S32, and Supplementary Figures S8–S11.

PERMANOVA revealed a significant effect of the state of consciousness on oscillatory activity (Subject_3_: F(3, 20) = 17.978, p < 0.001, R^2^ = 0.729; Subjectj_6_: F(3, 20) = 6.19, p < 0.001, R^2^ = 0.481; Subject_7_: F(3,20) = 10.374, p < 0.001, R^2^ = 0.609). For Subject 3, post-hoc analyses demonstrated a significant difference between FA and REM sleep (PERMANOVA, Subject_3_: F(1,10) = 9.252, p = 0.031, R^2^ = 0.481; Supplementary Table S33) while no significant differences were observed for Subjects 6 and 7 (PERMANOVA, Subject_6_: F(1,10) = 5.718, p = 0.227, R^2^ = 0.364; Subject_7_: F(1,10) = 3.282, p = 0.344, R^2^ = 0.247; Supplementary Tables S36 and S38). Subject 3 showed a significant reduction in delta relative power across all regions and an increase in frontal theta activity during FA compared to REM sleep (Table 4; Supplementary Table S34). Additionally, this subject exhibited increased alpha relative power in the frontal areas and reduced beta activity across widespread areas.

**Table 4:** Spectral power differences between false awakenings and standard sleep stages (PERMANOVA results).

Subject	Sleep Stage	Delta	Theta	Alpha	Beta	Low-Gamma
3	REM	↓ all regions	↑ frontal	↑ frontal	↑ frontal, central, temporal	**ns**
6	REM	ns	ns	ns	ns	**ns**
7	REM	ns	ns	ns	ns	**ns**
3	S1	ns	ns	ns	ns	↓ central
6	S1	↓ frontal	↓ central, temporal, parietal, occipital	↑ frontal, temporal, parietal, occipital	↓ central, temporal, parietal, occipital	↓ all regions
7	S1	ns	ns	ns	ns	ns
3	Wakefulness	↑ all regions	↑ all regions	↓ all regions	ns	↓ frontal, central, temporal
6	Wakefulness	ns	ns	ns	ns	ns
7	Wakefulness	↑ central	ns	↓ central	↓ central	↓ central

Summary of significant spectral power differences (PERMANOVA) between False Awakenings and three Standard Sleep Stages: REM sleep, Stage 1 sleep (S1), and wakefulness. Arrows indicate significant increases (↑) or decreases (↓) in relative power for each frequency band, with scalp regions specified. “ns” denotes non-significant comparisons. Common results across subjects are shown in bold.

Significant differences were also observed between FA and S1 for Subject 6 (PERMANOVA, F(1,10) = 9.575, p = 0.036, R^2^ = 0.489), whereas no significant differences were found for Subjects 3 and 7 (Subject_3_: PERMANOVA, F(1,10) = 0.780, p = 1.000, R^2^ = 0.072; Subject_7_: F(1,10) = 3.036, p = 0.286, R^2^ = 0.233). Specifically, Subject 6 exhibited a significant reduction in delta relative power in the frontal region, as well as reductions in theta and beta relative power across widespread areas (Supplementary Table S37). This subject also showed increased alpha and decreased low-gamma in several regions.

Subject 3 and 7 showed significant differences compared to wakefulness (PERMANOVA, Subject_3_: (F(1,10) = 15.087, p = 0.023, R^2^ = 0.601; Subject_7_: F(1,10) = 12.442, p = 0.020, R^2^ = 0.554), whereas Subject 6 did not (PERMANOVA, Subject_6_: F(1,10) = 3.069, p = 0.217, R^2^ = 0.235). Subject 3 exhibited significant increases in theta activity across all regions (Supplementary Table S35). Subjects 3 and 7 exhibited significant increases in delta activity across all recorded regions (Supplementary Tables S35 and S39). However, it is important to note that only the central region was recorded for Subject 7 (see Materials and Methods). Reductions in alpha relative power were observed across all regions in Subject 3, and in the central region in Subject 7. Both subjects also showed significant reductions in low-gamma activity, widespread in Subject 3 and restricted to the central region in Subject 7.

## Discussion

In this study, we investigated the neurophysiological bases of NOSC, such as LDs, SP, OBEs, and FAs. First, through PCA, we found that these ten NOSC differentiate from wakefulness, indicating that they exhibit distinct electrophysiological profiles. While conventional spectral analyses (e.g., mean PSD) might suggest that these experiences exist in an intermediate position between canonical stages of sleep and wakefulness^7,10,14^, the PCA results offer a more nuanced, structure-based view. In the reduced-dimensional space, these episodes showed no overlap with wakefulness and, in several cases, diverged not only from wakefulness but also from both REM sleep and S1.

Although some NOSC appear visually between canonical stages in the PCA projection (e.g., between REM sleep and S1 or Wakefulness and REM sleep), it is important to note that proximity in PCA space does not imply a transitional or hybrid physiological state. PCA organizes data based on shared variance in spectral features, and two states may appear close in the reduced space due to partial feature similarity, without being physiologically intermediate. In this study, the fact that these experiences form separate, compact clusters without overlap suggests that they are not merely extensions or blends of known stages, but may instead represent distinct neurophysiological configurations within the sleep spectrum. Further research is needed to test the robustness of this pattern across individuals and methodologies.

While NOSC share traits with both REM sleep and S1, they also exhibit specific differences. For example, LDs show a combination of delta and theta contributions, along with moderate involvement from alpha, beta and low-gamma. This pattern suggests that LDs share features with REM sleep while also exhibiting more active cortical modulation. However, PERMANOVA showed no significant differences between LDs and REM sleep. Baird et al. (2022) proposed that LDs occur within normal REM sleep; however, they found significant reductions in delta (2–4 Hz) and beta (12.5–35 Hz) power during LDs compared to REM sleep^8^. The difference between studies could be due to variations in the selection of segments for analysis. While we included only segments of phasic REM sleep (for REM sleep without consciousness) and incorporated the eye-mark in the LD period analyzed, they included both tonic and phasic REM sleep and excluded the eye-mark from the lucid segment studied. Thus, the inclusion of tonic REM sleep could be increasing delta power in the period of REM sleep without consciousness^30^.

Regarding SP we observed stronger influences from alpha, beta and low-gamma bands during the episodes. Furthermore, compared to REM sleep, both episodes exhibited reduced theta relative power and increased low-gamma activity. Both subjects reported full conscious awareness during SP, although only one successfully performed the eye movement marker. Our results partially replicate findings by Mainieri et al. (2021), who reported reduced theta and increased alpha during SP compared to REM sleep^14^. The authors introduced the term“lucid paralysis”to describe SP episodes characterized by heightened self-awareness and cognitive clarity, akin to LDs.

Regarding OBEs, they were primarily characterized by delta and theta frequency bands. Notably, only OBE_1_ exhibited a significant increase in delta relative power compared to REM sleep. Delta waves have been characterized according to their amplitude and context of occurrence. Unlike high-amplitude slow waves (75 μV) typically associated with generalized cortical deactivation^31^, lower amplitude delta activity (5–50 μV) has been interpreted as reflecting localized processes in primary sensory areas, potentially facilitating disconnection from the external environment^30,32^. In this context, the observed increase in delta activity during OBE_1_ may reflect a functional decoupling from external sensory input, supporting the immersive and internally driven nature of the experience. However, our analysis, based on PSD, does not allow us to determine their amplitude or spatial distribution. Further studies should address more directly in future using methodologies capable of characterizing wave morphology and localization in order to test this hypothesis.

Recently, more literature has emerged addressing the physiological correlates of OBEs. Weiler et al. (2025) reported an eye movement signal associated with OBEs; however, their data were limited to EOG and did not include EEG or sleep staging^33^. Campillo-Ferrer et al. (2025, preprint) reported EEG data from unusual bodily experiences (UBEs), including vestibular-motor sensations, tactile phenomena, distortions of body boundaries, lack of bodily sensations, and OBEs^34^. Their analyses comparing UBEs to baseline brain states (REM, NREM, or wakefulness during meditation) revealed significant decreases in delta and theta, alongside increases in beta and gamma activity. The discrepancies between their findings and ours may be due to differences in the episodes experienced by the participants. While they examined a heterogeneous set of unusual bodily experiences (UBEs), we focused specifically on OBEs, where subjects explicitly reported the perception of leaving the body, with vestibular and motor sensations accompanied by visual content. Furthermore, Campillo et al. included states occurring during wakefulness, NREM, and REM sleep, whereas in our study both OBEs occurred during REM sleep. Taken together, these differences underscore the need for a clearer differentiation between OBEs and other UBEs, as well as for systematic EEG investigations of OBEs occurring specifically during sleep.

FA states were predominantly shaped by alpha, beta, and low-gamma activity, with theta showing comparatively lower contributions. However, the contributions of these bands varied across the FA episodes with no clear pattern across subjects. Among the FA episodes, Subject 7 performed the ocular signal typically used to mark lucid experiences. Following the rationale proposed by Manieri et al. (2021), who described “lucid sleep paralysis” in cases where participants signaled lucidity during SP episodes, we suggest that this episode could represent a case of “lucid false awakening”. Interestingly, in the PCA space, the spectral profile of this FA episode closely resembled that of lucid dreaming. This similarity may reflect a shared underlying neural signature or conscious awareness processing. In contrast, the other two subjects experiencing FAs did not mark lucidity, and both reported awakening shortly after recognizing the nature of the episode. Thus, differences across FA episodes may reflect differences in lucidity, with Subject 7 potentially experiencing a “lucid FA”^14^. It is worth noting that we found partially similar results to Manieri et al. (2021), with a significant increase in alpha in Subject 3^14^.

Finally, our findings suggest that NOSC may differ meaningfully from wakefulness, despite occurring within established sleep stages. While most previous studies have focused on increased fast-frequency activity as a hallmark of conscious awareness during sleep^2,7,8,14,35^, our results also raise the possibility that delta activity could contribute to functional disconnection from the external world, supporting immersive dream-like experiences. Further work is needed to examine the specific role and morphology of these slow oscillations in NOSC.

### Limitations

A key limitation of this study is the variability in EEG segment selection for the NOSC, as some experiences were marked by clear behavioral signals (e.g., eye movements), while others relied on retrospective reports. In some cases, subjects provided an eye signal to mark the beginning, but the end of the experience was less clear; in other cases, the report was obtained immediately after awakening, so the beginning was unclear. Additionally, some EEG channels were removed from the analysis due to poor signal quality. One participant (Subject 7) was originally part of a separate nap study and was included after spontaneously performing the eye-movement signal. Unlike other participants, this subject did not complete the home-monitoring phase, and only a subset of EEG channels (C3, C4, Cz) was available, limiting comparability. Another methodological limitation is that electrode impedances were maintained below 10 kΩ. Although this threshold is within acceptable standards for EEG recordings, it may still allow for increased noise. This could compromise the signal-to-noise ratio, especially in higher frequency bands (e.g., beta and gamma), which are more susceptible to contamination by electrical and movement-related artifacts^36^. Finally, the sample size and number of episodes per state were limited, which may affect the generalizability and statistical robustness of the findings. However, it is important to note that studies on spontaneous NOSC are inherently difficult to conduct, and most existing work in this area is also based on small samples. We view this study as a step toward building a foundation for future investigations. Larger datasets and collaborative research efforts will be essential for validating and extending these findings.

## Materials & methods

### Participants

Participants were recruited via social media advertisements targeting individuals with experiences of LDs, SPs, OBEs or FAs during sleep. Eligibility criteria included experiencing at least one episode of LD, SP, OBE or FA per week, with no history of or diagnosed sleep disorders, psychiatric disorders, substance abuse, epilepsy, or migraines. Of the initial pool, ten participants (n = 10) met the eligibility criteria and, after successfully using the eye movement signal in at least two self-reported episodes during the home-monitoring phase, were invited to the sleep laboratory. All participants completed four nights of polysomnographic recordings. However, during the laboratory phase, four participants did not experience any NOSC. As a result, only six participants (n = 6) exhibited in-lab episodes (LDs, SPs, FAs or OBEs) and were included in the final analysis. We included in the sample one participant (Subject 7) who was originally part of a separate nap study conducted in the same laboratory. During that study, Subject 7 experienced a FA and as this subject was aware of the study and familiar with the eye-movement technique, left the eye mark. Given the relevance of this case, the participant was asked for consent to include their data in the present study, which was granted. Additionally, this participant was recorded during a nap rather than a full night, and only a limited set of EEG channels was available (C3, C4, Cz). These methodological differences are acknowledged in the study’s limitations. Additionally, this participant exhibited a sleep-onset REM period (SOREMP), which is suggestive of narcolepsy. While this participant had no formal diagnosis, future studies should include clinical screenings to better control for potential sleep disorders. Thus, the final sample consisted of 11 participants (n = 11, 5 female and 6 male), but only 7 participants (n = 7, 2 female and 5 male) were included in the analysis.

### Inclusion & ethics statement

The study was conducted in accordance with the ethical principles outlined in the Declaration of Helsinki. The protocol was reviewed and approved by the Biomedical Research Ethics Committee of the Alberto C. Taquini Institute for Translational Medicine Research (IATIMET) and the Human Ethics Committee, Faculty of Medicine, University of Buenos Aires (Comité de Ética Humana, Facultad de Ciencias Médicas, Universidad de Buenos Aires). All participants provided written informed consent before their inclusion in the study.

### Procedure

#### Initial screening and training

Participants were instructed to use a specific eye movement pattern (three consecutive left–right movements) to signal the occurrence of LDs, SP, OBEs and FAs. During a 15-day period of home monitoring, they documented their dreams and whether they had performed the eye signal, using structured dream diaries. The completion of the eye signal was self-reported. After this period, the researchers reviewed the diaries and quantified the number of episodes in which participants reported having used the eye signal. Only those who consistently reported using the signal in at least two separate episodes were invited to participate in the subsequent sleep laboratory study.

#### In-Lab procedure

In the sleep laboratory, participants were invited to complete four consecutive nights of polysomnographic recordings, during which they were allowed to sleep a full night according to their usual sleep schedule. During these nights, they were instructed to perform the eye-mark signal immediately upon becoming aware of a LD, SP, OBE, or FA. Participants were informed that the signal could be performed at any moment during the night upon experiencing conscious awareness during sleep. After each signal, participants were spontaneously awakened and asked to provide a verbal report of their experience, which was subsequently recorded. It is important to note that not all participants awakened spontaneously after each NOSC. In such cases, the end of the EEG segment was defined arbitrarily (see below). Reports were then evaluated by two independent raters to confirm whether the content matched the criteria for LD, SP, OBE, or FA.

### Experimental setting

All recordings were conducted in a sleep laboratory equipped with a comfortable, sound-attenuated room for the participant. The room included a bed, a bedside table with a lamp, a small desk, and the necessary recording equipment. Air conditioning was provided to ensure thermal comfort. There were no video cameras present in the room to preserve the participant’s privacy. The experimenter monitored the polysomnographic signals in real time from an adjacent control room and the audio of the dream reports were recorded. Upon awakening from a NOSC (LD, SP, OBE, or FA), participants were instructed to immediately report the event, regardless of the time during the night.

### Polysomnography

Polysomnographic recordings were obtained using electroencephalography systems (BrainAmp DC, 32 channels, Brain Vision), including electroencephalography (EEG), electromyography (EMG), and electrooculography (EOG). EEG electrodes were placed at F3, F4, F7, F8, Fz, C3, C4, Cz, T3, T4, T5, T6, P3, P4, Pz, O1, and O2, according to the International 10–20 system, referenced to electrodes attached to both mastoids. However, for Subject 7, only C3, C4, and Cz were available due to the recording setup of the original study in which they participated. The EOG activity was recorded using two electrodes: one placed 1 cm above the right eyebrow and another 1 cm below the left eye. Data were sampled at 250 Hz, with electrode impedance maintained below 10 kΩ. Sleep stages were manually scored following standard criteria^37^. Scoring was performed visually by an experienced rater using EEG, EOG, and EMG signals. Manual artifact correction was performed by visually inspecting all EEG recordings. Segments that showed clear signs of artifacts, such as muscle activity, electrode noise, or movement, were excluded from the analysis.

### Classification of non-ordinary states of consciousness

The classification of each reported experience into one of the target phenomena (LDs, SP, OBEs or FAs) was based on first-person reports (Supplementary Table S40). Two trained researchers independently reviewed the content of the verbal report. Only episodes in which both raters agreed on the appropriateness of the classification were included in the final dataset. Classification was grounded in predefined phenomenological criteria. LDs were identified when participants reported being aware that they were dreaming during the ongoing dream experience^2,4^. SP was defined by the report of a vivid sensation of bodily paralysis, characterized by the inability to initiate voluntary movements^10–12^. OBEs were defined by the sensation of separation from the physical body, accompanied by the perception of the environment from an external viewpoint^19,38,39^. FAs were classified when participants described the compelling impression of having woken up (typically in the laboratory setting) followed by the realization that they were still dreaming^14,28^.

### Selection of EEG segments

In the best-case scenarios, the onset of the EEG segments corresponding to NOSC was clearly marked by the predefined eye-movement signal, and the offset by spontaneous awakening at the end of the episode (Subject 3’s LD, OBE_1_, Subject 5’s SP, Subject 7’s FA). In contrast, when participants provided the eye-movement marker but did not wake up immediately after the experience (Subjects 1 and 2), a fixed 30-s segment was selected starting at the marker. This duration was chosen based on the episodes where both onset and offset were (eye mark plus awakening), which consistently lasted over 30 s, ensuring that the selected segment would reliably capture the experience itself. Finally, when no eye-movement marker was available but the participant reported a NOSC immediately upon awakening (Subjects 3 and 6’s FA), a 30-s segment preceding the awakening was selected. This approach of selecting arbitrary segments based on subjects’ reports, was consistent with prior literature, where retrospective alignment to awakening is commonly used when no behavioral marker is present^14^. An exception was made for Subject 4’s SP and Subject 3’s OBE_2_. In the case of Subject 4, the participant briefly woke up, went back to sleep, and then reported entering the episode immediately after falling asleep, which ended upon awakening (approximately 60 s later). The report of SP was provided immediately after this final awakening. Although this segment exceeded the standard 30-s duration, its inclusion was justified by the participant’s detailed report and the lack of a behavioral marker. Similarly, in the case of Subject 3’s OBE_2_, the participant experienced multiple brief awakenings during the night, and the only REM sleep period recorded corresponded to the reported OBE episode. The experience was reported immediately upon awakening, and, as in the SP case, its inclusion was justified by the detailed report and absence of clear behavioral markers.

Control REM sleep segments were selected based on temporal proximity and the absence of artifacts. Phasic REM sleep segments most temporally distant from the target experience were prioritized for comparison. The NOSC was used as the temporal reference, and the duration of each control segment was matched to that of the episode. As mentioned before, an exception was Subject 3’s OBE_2_, where the episode was followed by awakening, but no additional REM sleep segment without conscious awareness content was available within the same night. In this case, a control REM sleep segment of matching duration and characteristics (phasic REM sleep) was selected from a different night in which no NOSC were reported. While cross-night comparisons are not ideal, this was the only viable option to enable within-subject analysis. Stage 1 sleep (S1) and wakefulness segments were selected according to the same criteria (Supplementary Figure S11). A summary of segment definitions for each case is provided in Table 5.

**Table 5:** Details of each subject’s data processing.

Subject	Type of NOSC	Removed Channels	Eye signaling (onset of the NOSC)	EEG segment definition (offset of the NOSC)	Duration of the NOSC
1	Lucid Dream	Fz, F8, C3, C4, T5	Yes	Arbitrary 30 s segment	30 s
2	Lucid Dream	F4, F7, C3, C4, T3, T4, T5, T6, P3, Pz, P4, O1, O2	Yes	Arbitrary 30 s segment	30 s
3	Lucid Dream	C3, C4, Cz, T6, Pz, P4, O1, O2	Yes	Arbitrary 30 s segment	30 s
	OBE_1_	F3, Fz, C3, Cz, P4, O1	Yes	30 s before reported awakening	57 s
	OBE_2_	T3, T4, T5, T6, P3, Pz, O1	No	Segment ended upon awakening	173 s
	False Awakening	F4, T5	No	30 s before reported awakening	30 s
4	Sleep Paralysis	T4, T6, P3, P5	No	Segment ended upon awakening	87 s
5	Sleep Paralysis	F8, T6, O2	Yes	Segment ended upon awakening	75 s
6	False Awakening	None	No	30 s before reported awakening	30 s
7	False Awakening	None (only C3, C4, Cz available)	Yes	Segment ended upon awakening	32 s

Details of Each Subject’s Data Processing. Summary of data preprocessing for each subject, including the type of experience analyzed, removed EEG channels due to artifacts, availability of eye signaling, and criteria used to define the EEG segment for analysis. EEG segments were either defined arbitrarily (30 s), based on reported awakening, or extended until awakening when possible.

### EEG data preprocessing

The EEG signals were filtered using a 1 Hz high-pass filter to eliminate respiratory artifacts, a 45 Hz low-pass filter, and a 50 Hz notch filter. Then, the EEG data were preprocessed using Independent Component Analysis (ICA) to correct ocular artifacts. ICA was performed using the MNE Python library^40^ with the *mne.preprocessing.ICA* function. The procedure involved decomposing the EEG signals into a specified number of components. The selection of components to be removed was determined subjectively, based on visual inspection of the independent components.

### EEG spectral analysis and area under the curve (AUC)

Each NOSC and sleep stage segment was divided into six equal-length, non-overlapping temporal segments. To analyze the spectral properties of the EEG segments, they were divided into consecutive windows of 312 samples with 50% overlap. For each window, the power spectral density (PSD) was estimated using Welch’s method with a Hanning window to minimize spectral leakage. The PSD was computed for five frequency bands of interest: delta (1–4 Hz), theta (4–8 Hz), alpha (8–13 Hz), beta (13–30 Hz), and low-gamma (30–45 Hz), and averaged across five scalp regions (frontal, central, temporal, parietal, occipital). All analyses were conducted using the MNE-Python library^40^. The area under the curve (AUC) for each frequency band was calculated across all segments using numerical integration via the trapezoidal rule (*numpy.trapz* function from the NumPy Python library^41^). AUCs were then normalized to obtain relative AUC values, ensuring comparability between each NOSC and REM sleep, S1 and wakefulness.

### Principal component analysis (PCA)

To investigate patterns in spectral EEG data and reduce its dimensionality, we applied Principal Component Analysis (PCA). This technique linearly transforms a set of potentially correlated spectral variables into a new set of orthogonal principal components, which are ordered by the proportion of variance they explain. Separate PCAs were conducted for each NOSC. Each observation in the analysis corresponded to a time segment from a specific brain state (e.g., lucid dreaming, REM sleep, S1, or wakefulness), and each variable represented relative spectral power within a specific frequency band and scalp region (e.g., delta-frontal, theta-central). To interpret the resulting components, we focused on three metrics that capture complementary aspects of the relationship between the original variables and the component space. First, we computed the correlation between each spectral variable and the principal components. These values reflect the strength and direction of association between a given variable and a component and are visualized in biplots as arrows, where the arrow direction indicates the axis along which the variable increases, and the length reflects the magnitude of the correlation. Second, we calculated the contribution of each variable to each component. Expressed as a percentage, this metric quantifies how much each variable contributes to the construction of a given principal component. Higher contribution values indicate variables that play a greater role in defining the structure of that component. Third, we computed the squared cosine (cos^2^) values, which quantify the quality of representation of a variable in the reduced-dimensional space. Specifically, cos^2^ measures the proportion of the variance of a given variable that is captured by the component axes. High cos^2^ values indicate that a variable is well represented in the selected component plane and that its position in the biplot reliably reflects its behavior in the original data space.

We visualized PCA results using biplots, where points represent individual time segments and are color-coded by state of consciousness, while arrows represent spectral variables. The spatial layout of arrows in the biplot conveys their correlation structure: closely aligned arrows represent positively correlated variables, while perpendicular ones reflect orthogonality. In addition, the projection of each variable’s arrow onto a component axis provides a geometric representation of its correlation and contribution to that axis. While PCA projections can suggest proximity between brain states in terms of shared spectral features, such spatial relations should not be interpreted as evidence of transitional or hybrid states. The presence of separate and non-overlapping clusters in the reduced-dimensional space supports the idea that these experiences possess internally consistent and structurally distinct profiles, rather than merely occupying a point along a continuum between canonical stages.

This multivariate, data-driven approach enabled us to identify frequency-band and region-specific spectral features that distinguish between NOSC. By jointly examining correlation, contribution, and cos^2^, we could assess both the structural importance and representational fidelity of each variable in the reduced PCA space. All analyses were conducted in R^42^ using the *FactoMineR* package^43^, with visualization and interpretation supported by *factoextra*^44^.

### Permutation-based multivariate analysis of variance (PERMANOVA)

To assess differences in EEG spectral profiles across states of consciousness, we applied permutation-based multivariate analysis of variance (PERMANOVA). The analysis was performed in R^42^ using the *vegan* package^45^. For each subject, six time segments per NOSC were included as observations, corresponding to those defined in the spectral area-under-the-curve (AUC) computation (see Spectral Analysis section). Each segment was characterized by relative spectral power values across five frequency bands (delta, theta, alpha, beta, low-gamma) and five scalp regions (frontal, central, temporal, parietal, occipital), resulting in up to 25 features per observation (with possible variation due to channel loss in specific cases).

We implemented three complementary levels of PERMANOVA analysis to test for state-related effects with increasing specificity. First, a global PERMANOVA was conducted for each subject to test for overall spectral differences across the four experimental conditions: the target NOSC (e.g., lucid dreaming) and three baseline states (REM sleep, S1, and wakefulness). This resulted in one model per subject (10 in total), each using all available spectral and spatial variables simultaneously. The main effect of state was tested using the *adonis2* function in R with 9,999 permutations. Assumptions of homogeneous multivariate dispersions were evaluated using *betadisper*. Second, we performed pairwise post hoc PERMANOVA comparisons between the target NOSC and each of the three baseline conditions (e.g., LD vs REM, LD vs S1, LD vs wakefulness). These contrasts were performed within each subject’s dataset using the *pairwise.adonis* function, with Bonferroni correction applied across comparisons. This step identified specific state pairs responsible for the global effects observed. Third, to explore the spatiotemporal specificity of effects, we conducted exploratory univariate PERMANOVAs within each frequency band and scalp region separately. These models allowed us to localize differences in spectral power to specific frequency–region combinations and provided insight into the anatomical and spectral specificity of consciousness-related effects. To account for multiple comparisons, FDR-adjusted p-values (Benjamini–Hochberg method) are reported. All analyses used 9,999 permutations to ensure robust estimation of p-values. Together, this multistep approach allowed us to examine both the overall multivariate structure of the spectral data and its underlying regional and frequency-specific components.

### Use of large language models (LLMs)

During the manuscript preparation, a Large Language Model (ChatGPT, OpenAI 4.0) was used to assist with English language editing and grammar correction. The authors carefully reviewed and verified all LLM-assisted edits to ensure the accuracy and integrity of the final text.

## Software and code availability

Data acquisition was performed using BrainVision Recorder 2.0 (Brain Products GmbH, Germany). Data analyses were conducted using publicly available packages, including MNE-Python, FactoMineR, and vegan (adonis2 function). Analysis scripts will be made available in an online repository after publication.

## Supplementary Information

Supplementary Information.

## Data Availability

The authors declare that data supporting the findings of this study are available from the corresponding author upon request.
