# Supplementary material for: Exploratory study of non-ordinary states of consciousness during sleep show distinct electrophysiological features from wakefulness and canonical sleep stages
Source: Sci Rep. 2025 Sep 29;15:33586. doi: 10.1038/s41598-025-18748-7 (PMC12479757; doi:10.1038/s41598-025-18748-7)
Supplement: Supplementary file 1 — Supplementary Information. [file 41598_2025_18748_MOESM1_ESM.docx]

**Supplementary Material**

**Exploratory Study of Non-Ordinary States of Consciousness During Sleep Show Distinct Electrophysiological Features from Wakefulness and Canonical Sleep Stages**

Nerea L. Herrero, Yohann Corfdir, Aylin A. Vázquez-Chenlo,

Lucila Capurro & Cecilia Forcato^#^.

Laboratorio de Sueño y Memoria, Departamento de Ciencias de la Vida, Instituto Tecnológico de Buenos Aires (ITBA), Iguazú 341, (1437) Capital Federal, Buenos Aires, Argentina

**Supplementary Material Overview**

This supplementary material provides a detailed presentation of the principal component analysis (PCA) and PERMANOVA results that complement the findings of the main manuscript. The content is organized by non-ordinary state of consciousness (NOSC): lucid dreaming (LD), sleep paralysis (SP), out-of-body experiences (OBE), and false awakenings (FA). For each state, the results are grouped into two sections. The first section presents the PCA results for all subjects, including tables summarizing the contribution and squared cosine (cos²) values of each condition (NOSC, REM sleep, S1, and wakefulness) to the first two principal components, along with figures displaying the contribution of spectral variables to each dimension with color-coded correlation signs. The second section includes the PERMANOVA results, comparing spectral profiles between conditions across subjects.

In addition to the multivariate analyses, this supplement includes two complementary elements: (1) a table presenting the first-person reports corresponding to each of the ten episodes analyzed in the study, which served as the basis for their phenomenological classification, and (2) a figure displaying illustrative 30-second EOG/EEG/EMG segments from REM sleep, Stage 1 (S1), and wakefulness, representing the canonical conditions used as comparison baselines throughout the main analyses.

**Lucid Dreaming**

**PCA Results**

**Subject 1**

| **Supplementary Table S1** Subject 1 – Principal Component Analysis: Contribution and Representation of Conditions | | | | |
| --- | --- | --- | --- | --- |
| Condition | % Contribution PC 1 | % Contribution PC 2 | Cos² PC 1 | Cos² PC 2 |
| LD | 10.802 | 13.221 | 0.440 | 0.150 |
| REM sleep | 10.461 | 29.745 | 0.342 | 0.294 |
| S1 | 7.382 | 50.385 | 0.204 | 0.290 |
| Wakefulness | 71.354 | 6.649 | 0.925 | 0.032 |
| **Supplementary Table S1** Principal Component Analysis results for Subject 1. The table shows the percentage of contribution and squared cosine (cos²) values for each condition across the first two principal components (PC1 and PC2). | | | | |


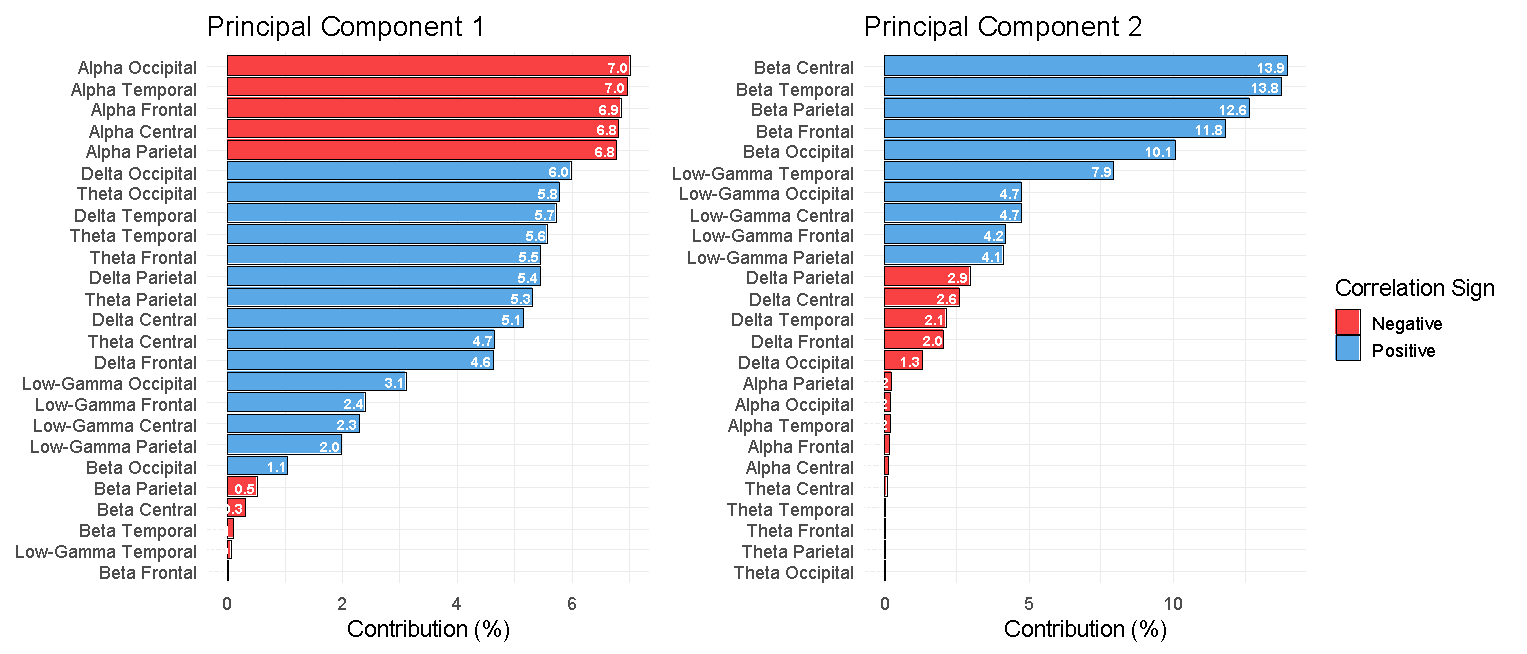


**Supplementary Figure S1** Contributions of EEG spectral variables to the first two principal components in Subject 1. Each bar represents a variable defined by a frequency band and cortical region, with bar length indicating its percentage contribution to the corresponding component. Colors represent the sign of the correlation between the variable and the component: blue for positive and red for negative. Variables are ordered by contribution within each component.

**Subject 2**

| **Supplementary Table S2** Subject 2 – Principal Component Analysis: Contribution and Representation of Conditions | | | | |
| --- | --- | --- | --- | --- |
| Condition | % Contribution PC 1 | % Contribution PC 2 | Cos² PC 1 | Cos² PC 2 |
| LD | 12.854 | 11.181 | 0.443 | 0.118 |
| REM sleep | 23.901 | 7.324 | 0.660 | 0.068 |
| S1 | 4.549 | 64.614 | 0.181 | 0.586 |
| Wakefulness | 58.696 | 16.880 | 0.688 | 0.071 |
| **Supplementary Table S2** Principal Component Analysis results for Subject 2. The table shows the percentage of contribution and squared cosine (cos²) values for each condition across the first two principal components (PC1 and PC2). | | | | |

**
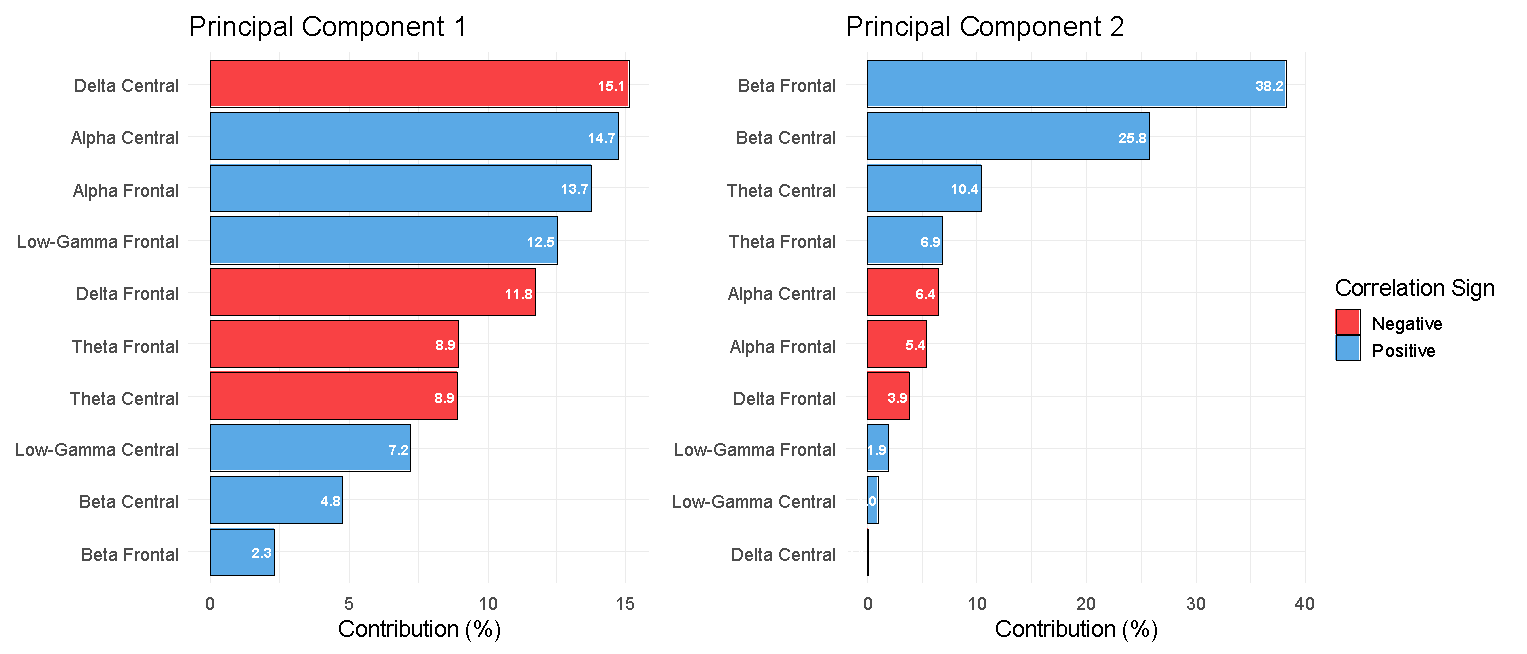
**

**Supplementary Figure S2** Contributions of EEG spectral variables to the first two principal components in Subject 2. Each bar represents a variable defined by a frequency band and cortical region, with bar length indicating its percentage contribution to the corresponding component. Colors represent the sign of the correlation between the variable and the component: blue for positive and red for negative. Variables are ordered by contribution within each component.

**Subject 3**

| **Supplementary Table S3** Subject 3 – Principal Component Analysis: Contribution and Representation of Conditions | | | | |
| --- | --- | --- | --- | --- |
| Condition | % Contribution PC 1 | % Contribution PC 2 | Cos² PC 1 | Cos² PC 2 |
| LD | 12.944 | 38.610 | 0.242 | 0.212 |
| REM sleep | 21.533 | 43.155 | 0.519 | 0.284 |
| S1 | 6.545 | 9.968 | 0.363 | 0.175 |
| Wakefulness | 58.978 | 8.267 | 0.880 | 0.041 |
| **Supplementary Table S3** Principal Component Analysis results for Subject 3. The table presents the percentage of contribution and squared cosine (cos²) values for each condition across the first two principal components (PC1 and PC2). | | | | |


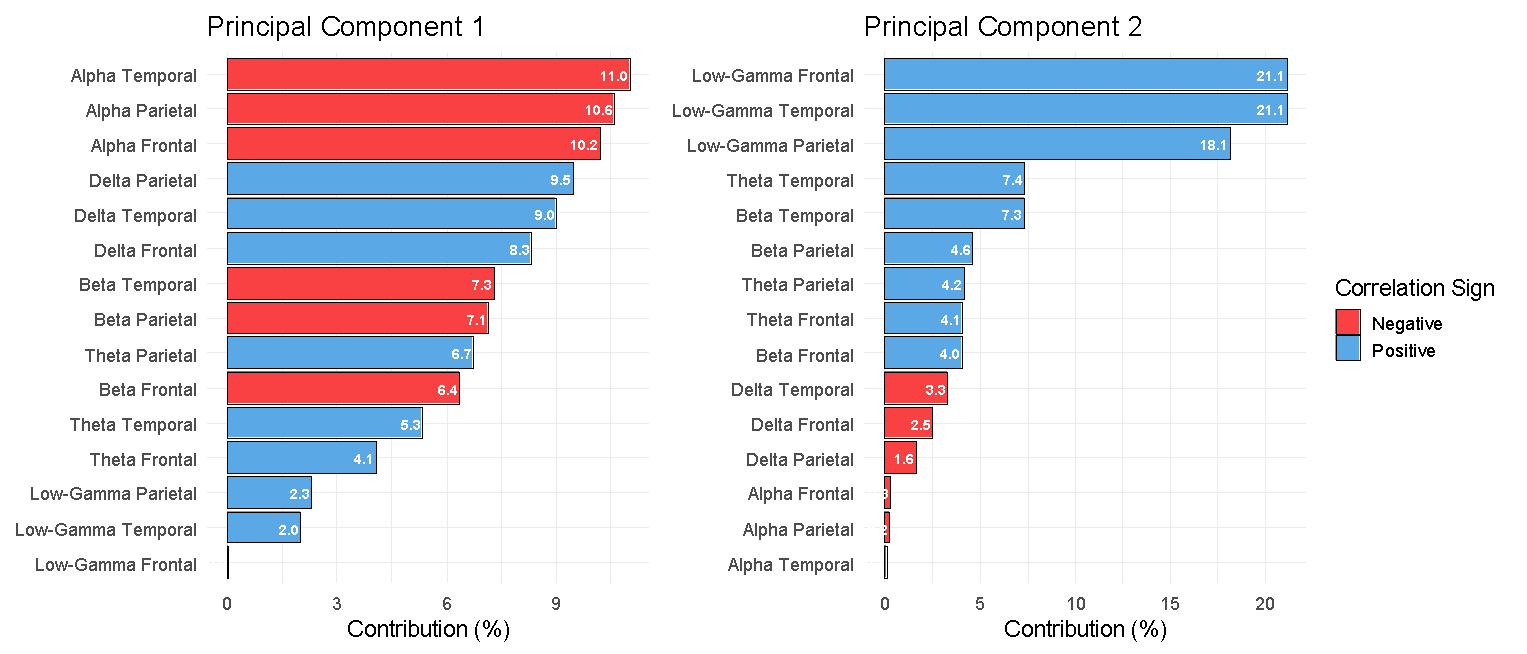


**Supplementary Figure S3** Contributions of EEG spectral variables to the first two principal components in Subject 3. Each bar represents a variable defined by a frequency band and cortical region, with bar length indicating its percentage contribution to the corresponding component. Colors represent the sign of the correlation between the variable and the component: blue for positive and red for negative. Variables are ordered by contribution within each component.

**PERMANOVA Results**

**Subject 1**

| **Supplementary Table S4** Subject 1 – Post hoc results comparing sleep states | | | | | | |
| --- | --- | --- | --- | --- | --- | --- |
| Comparison | Df | Sums Of Squares | F-value | R² | p-value | Adjusted p-value |
| Lucid Dream vs REM sleep | 1 | 0.008 | 0.563 | 0.054 | 0.704 | 1.000 |
| Lucid Dream vs S1 | 1 | 0.041 | 3.244 | 0.245 | 0.036 | 0.217 |
| Lucid Dream vs Wakefulness | 1 | 0.787 | 77.596 | 0.886 | 0.002 | 0.013 |
| REM sleep vs Wakefulness | 1 | 0.843 | 94.868 | 0.905 | 0.002 | 0.013 |
| S1 vs REM sleep | 1 | 0.068 | 5.931 | 0.372 | 0.004 | 0.023 |
| S1 vs Wakefulness | 1 | 0.710 | 99.881 | 0.909 | 0.003 | 0.016 |
| **Supplementary Table S4** Post hoc PERMANOVA results comparing Lucid Dreaming, REM sleep, S1, and Wakefulness for Subject 1. Bonferroni-adjusted p-values are reported. | | | | | | |

| **Supplementary Table S5** Spectral power differences between Lucid Dreaming and Wakefulness for Subject 1 | | | | | |
| --- | --- | --- | --- | --- | --- |
| Frequency Band | Brain Region | F-value | R² | p-value | Adjusted p-value |
| Delta | Frontal | 74.791 | 0.882 | 0.002 | 0.004 |
|  | Central | 45.757 | 0.821 | 0.002 | 0.004 |
|  | Temporal | 122.019 | 0.924 | 0.002 | 0.004 |
|  | Parietal | 33.164 | 0.768 | 0.001 | 0.004 |
|  | Occipital | 41.451 | 0.806 | 0.002 | 0.004 |
| Theta | Frontal | 49.154 | 0.831 | 0.002 | 0.004 |
|  | Central | 14.808 | 0.597 | 0.003 | 0.004 |
|  | Temporal | 68.777 | 0.873 | 0.002 | 0.004 |
|  | Parietal | 38.106 | 0.792 | 0.002 | 0.004 |
|  | Occipital | 64.802 | 0.866 | 0.002 | 0.004 |
| Alpha | Frontal | 393.831 | 0.975 | 0.003 | 0.004 |
|  | Central | 121.754 | 0.924 | 0.002 | 0.004 |
|  | Temporal | 322.076 | 0.970 | 0.002 | 0.004 |
|  | Parietal | 104.899 | 0.913 | 0.002 | 0.004 |
|  | Occipital | 426.070 | 0.977 | 0.002 | 0.004 |
| Beta | Frontal | 0.198 | 0.019 | 0.671 | 0.699 |
|  | Central | 0.289 | 0.028 | 0.619 | 0.673 |
|  | Temporal | 0.006 | 0.001 | 0.932 | 0.932 |
|  | Parietal | 0.330 | 0.032 | 0.551 | 0.627 |
|  | Occipital | 5.115 | 0.338 | 0.019 | 0.024 |
| Low-Gamma | Frontal | 29.245 | 0.745 | 0.003 | 0.004 |
|  | Central | 19.194 | 0.657 | 0.005 | 0.007 |
|  | Temporal | 6.747 | 0.403 | 0.040 | 0.048 |
|  | Parietal | 30.822 | 0.755 | 0.002 | 0.004 |
|  | Occipital | 29.781 | 0.749 | 0.003 | 0.004 |
| **Supplementary Table S5** PERMANOVA results comparing Lucid Dreaming and Wakefulness across frequency bands and brain regions for Subject 1. FDR-adjusted p-values (Benjamini-Hochberg method) are reported. | | | | | |

**Subject 2**

| **Supplementary Table S6** Subject 2 – Post hoc results comparing sleep states | | | | | | |
| --- | --- | --- | --- | --- | --- | --- |
| Comparison | Df | Sum Of Squares | F-value | R² | p-value | Adjusted p-value |
| Lucid Dream vs REM sleep | 1 | 0.015 | 1.597 | 0.138 | 0.2075 | 1.000 |
| Lucid Dream vs S1 | 1 | 0.131 | 12.345 | 0.552 | 0.0028 | 0.017 |
| Lucid Dream vs Wakefulness | 1 | 0.711 | 46.144 | 0.822 | 0.0022 | 0.013 |
| REM sleep vs Wakefulness | 1 | 0.751 | 46.450 | 0.823 | 0.0022 | 0.013 |
| S1 vs REM sleep | 1 | 0.111 | 9.727 | 0.493 | 0.0043 | 0.026 |
| S1 vs Wakefulness | 1 | 0.498 | 28.843 | 0.743 | 0.002 | 0.012 |
| **Supplementary Table S6** Post hoc PERMANOVA results comparing Lucid Dreaming, REM sleep, S1, and Wakefulness for Subject 2. Bonferroni-adjusted p-values are reported. | | | | | | |

| **Supplementary Table S7** Spectral power differences between Lucid Dreaming and S1 for Subject 2 | | | | | |
| --- | --- | --- | --- | --- | --- |
| Frequency Band | Brain Region | F-value | R² | p-value | Adjusted p-value |
| Delta | Frontal | 24.755 | 0.712 | 0.001 | 0.011 |
|  | Central | 8.841 | 0.469 | 0.021 | 0.043 |
| Theta | Frontal | 1.689 | 0.145 | 0.214 | 0.278 |
|  | Central | 0.645 | 0.061 | 0.447 | 0.497 |
| Alpha | Frontal | 4.752 | 0.322 | 0.057 | 0.095 |
|  | Central | 2.203 | 0.181 | 0.159 | 0.231 |
| Beta | Frontal | 15.908 | 0.614 | 0.004 | 0.011 |
|  | Central | 14.779 | 0.596 | 0.004 | 0.011 |
| Low-Gamma | Frontal | 6.017 | 0.376 | 0.002 | 0.011 |
|  | Central | 0.097 | 0.010 | 0.761 | 0.764 |
| **Supplementary Table S7** PERMANOVA results comparing Lucid Dreaming and S1 across frequency bands and brain regions for Subject 2. FDR-adjusted p-values (Benjamini-Hochberg method) are reported. | | | | | |

| **Supplementary Table S8** Spectral power differences between Lucid Dreaming and Wakefulness for Subject 2 | | | | | |
| --- | --- | --- | --- | --- | --- |
| Frequency Band | Brain Region | F-value | R² | p-value | Adjusted p-value |
| Delta | Frontal | 17.062 | 0.630 | 0.004 | 0.006 |
|  | Central | 90.661 | 0.901 | 0.002 | 0.005 |
| Theta | Frontal | 11.819 | 0.542 | 0.005 | 0.008 |
|  | Central | 24.215 | 0.708 | 0.002 | 0.005 |
| Alpha | Frontal | 24.197 | 0.708 | 0.003 | 0.005 |
|  | Central | 140.884 | 0.934 | 0.002 | 0.005 |
| Beta | Frontal | 7.722 | 0.436 | 0.019 | 0.025 |
|  | Central | 5.061 | 0.336 | 0.051 | 0.056 |
| Low-Gamma | Frontal | 13.609 | 0.576 | 0.002 | 0.005 |
|  | Central | 0.994 | 0.090 | 0.332 | 0.336 |
| **Supplementary Table S8** PERMANOVA results comparing Lucid Dreaming and Wakefulness across frequency bands and brain regions for Subject 2. FDR-adjusted p-values (Benjamini-Hochberg method) are reported. | | | | | |

**Subject 3**

| **Supplementary Table S9** Subject 3 – Post hoc results comparing sleep states | | | | | | |
| --- | --- | --- | --- | --- | --- | --- |
| Comparison | Df | Sum Of Squares | F-value | R² | p-value | Adjusted p-value |
| Lucid Dream vs REM sleep | 1 | 0.096 | 2.534 | 0.202 | 0.087 | 0.522 |
| S1 vs Lucid Dream | 1 | 0.069 | 2.303 | 0.187 | 0.083 | 0.500 |
| Lucid Dream vs Wakefulness | 1 | 0.472 | 15.275 | 0.604 | 0.002 | 0.013 |
| REM sleep vs Wakefulness | 1 | 0.999 | 68.220 | 0.872 | 0.002 | 0.013 |
| S1 vs REM sleep | 1 | 0.052 | 3.821 | 0.276 | 0.019 | 0.113 |
| S1 vs Wakefulness | 1 | 0.758 | 114.559 | 0.920 | 0.003 | 0.016 |
| **Supplementary Table S9** Post hoc PERMANOVA results comparing Lucid Dreaming, REM sleep, S1, and Wakefulness for Subject 3. Bonferroni-adjusted p-values are reported. | | | | | | |

| **Supplementary Table S10** Spectral power differences between Lucid Dreaming and Wakefulness for Subject 3 | | | | | |
| --- | --- | --- | --- | --- | --- |
| Frequency Band | Brain Region | F-value | R² | p-value | Adjusted p-value |
| Delta | Frontal | 15.128 | 0.602 | 0.002 | 0.010 |
|  | Temporal | 10.586 | 0.514 | 0.001 | 0.010 |
|  | Parietal | 8.569 | 0.461 | 0.004 | 0.010 |
| Theta | Frontal | 6.346 | 0.388 | 0.020 | 0.031 |
|  | Temporal | 11.308 | 0.531 | 0.016 | 0.031 |
|  | Parietal | 10.760 | 0.518 | 0.013 | 0.029 |
| Alpha | Frontal | 52.793 | 0.841 | 0.002 | 0.010 |
|  | Temporal | 27.823 | 0.736 | 0.002 | 0.010 |
|  | Parietal | 12.338 | 0.552 | 0.008 | 0.025 |
| Beta | Frontal | 2.645 | 0.209 | 0.144 | 0.153 |
|  | Temporal | 3.779 | 0.274 | 0.072 | 0.088 |
|  | Parietal | 5.467 | 0.353 | 0.026 | 0.041 |
| Low-Gamma | Frontal | 0.231 | 0.023 | 0.766 | 0.768 |
|  | Temporal | 3.708 | 0.271 | 0.029 | 0.042 |
|  | Parietal | 2.454 | 0.197 | 0.141 | 0.153 |
| **Supplementary Table S10** PERMANOVA results comparing Lucid Dreaming and Wakefulness across frequency bands and brain regions for Subject 3. FDR-adjusted p-values (Benjamini-Hochberg method) are reported. | | | | | |

**Sleep Paralysis**

**PCA Results**

**Subject 4**

| **Supplementary Table S11** Subject 4 – Principal Component Analysis: Contribution and Representation of Conditions | | | | |
| --- | --- | --- | --- | --- |
| Condition | % Contribution PC 1 | % Contribution PC 2 | Cos² PC 1 | Cos² PC 2 |
| SP | 8.355 | 68.268 | 0.141 | 0.420 |
| REM sleep | 17.746 | 5.625 | 0.498 | 0.073 |
| S1 | 15.435 | 3.082 | 0.415 | 0.039 |
| Wakefulness | 58.464 | 23.026 | 0.666 | 0.127 |
| **Supplementary Table S11** Principal Component Analysis results for Subject 4. The table presents the percentage of contribution and squared cosine (cos²) values for each condition across the first two principal components (PC1 and PC2). | | | | |

*
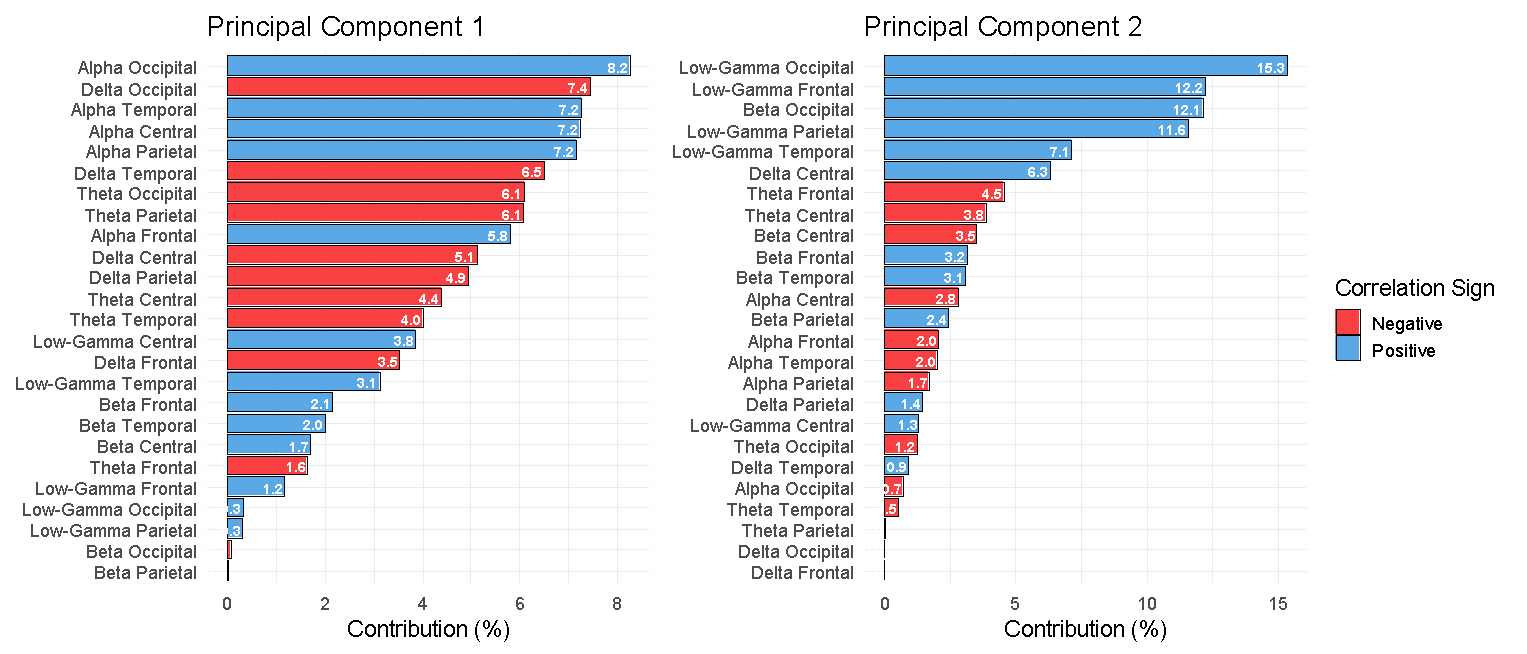
*

**Supplementary Figure S4** Contributions of EEG spectral variables to the first two principal components in Subject 4. Each bar represents a variable defined by a frequency band and cortical region, with bar length indicating its percentage contribution to the corresponding component. Colors represent the sign of the correlation between the variable and the component: blue for positive and red for negative. Variables are ordered by contribution within each component.

**Subject 5**

| **Supplementary Table S12.** Subject 5 – Contribution and Representation of Conditions | | | | |
| --- | --- | --- | --- | --- |
| Condition | % Contribution PC 1 | % Contribution PC 2 | Cos² PC 1 | Cos² PC 2 |
| SP | 4.781 | 16.209 | 0.184 | 0.239 |
| REM sleep | 29.163 | 10.100 | 0.798 | 0.071 |
| S1 | 14.375 | 26.449 | 0.58 | 0.179 |
| Wakefulness | 51.682 | 47.243 | 0.758 | 0.127 |
| **Supplementary Table S12** Principal Component Analysis results for Subject 5. The table presents the percentage of contribution and squared cosine (cos²) values for each condition across the first two principal components (PC1 and PC2). | | | | |

**
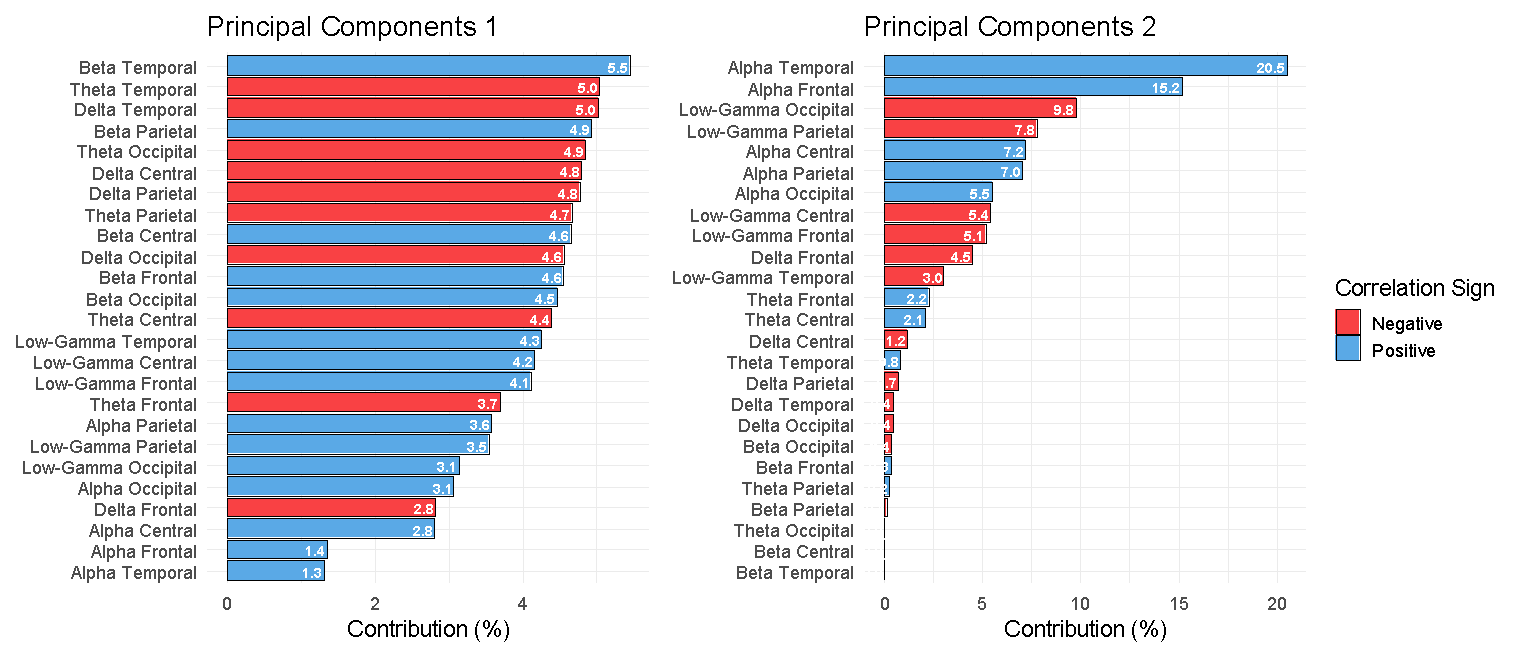
**

**Supplementary Figure S5** Contributions of EEG spectral variables to the first two principal components in Subject 5. Each bar represents a variable defined by a frequency band and cortical region, with bar length indicating its percentage contribution to the corresponding component. Colors represent the sign of the correlation between the variable and the component: blue for positive and red for negative. Variables are ordered by contribution within each component.

**PERMANOVA Results**

**Subject 4**

| **Supplementary Table S13** Subject 4 – Post hoc results comparing sleep states. | | | | | | |
| --- | --- | --- | --- | --- | --- | --- |
| Comparison | Df | Sum Of Squares | F-value | R² | p-value | Adjusted p-value |
| Sleep Paralysis vs REM sleep | 1 | 0.053 | 5.637 | 0.361 | 0.002 | 0.011 |
| Sleep Paralysis vs S1 | 1 | 0.055 | 5.462 | 0.353 | 0.007 | 0.042 |
| Sleep Paralysis vs Wakefulness | 1 | 0.312 | 21.756 | 0.685 | 0.003 | 0.016 |
| REM sleep vs Wakefulness | 1 | 0.378 | 36.783 | 0.786 | 0.002 | 0.010 |
| S1 vs REM sleep | 1 | 0.018 | 2.900 | 0.225 | 0.030 | 0.179 |
| S1 vs Wakefulness | 1 | 0.399 | 36.308 | 0.784 | 0.002 | 0.014 |
| **Supplementary Table** **S13** Post hoc PERMANOVA results comparing Sleep Paralysis, REM sleep, S1, and Wakefulness for Subject 4. Bonferroni-adjusted p-values are reported. | | | | | | |

| **Supplementary Table S14** Spectral power differences between Sleep Paralysis and REM sleep for Subject 4. | | | | | |
| --- | --- | --- | --- | --- | --- |
| Frequency Band | Brain Region | F-value | R² | p-value | Adjusted p-value |
| Delta | Frontal | 0.188 | 0.018 | 0.672 | 0.795 |
|  | Central | 15.815 | 0.613 | 0.006 | 0.030 |
|  | Temporal | 0.111 | 0.011 | 0.709 | 0.803 |
|  | Parietal | 0.599 | 0.056 | 0.485 | 0.651 |
|  | Occipital | 0.085 | 0.008 | 0.784 | 0.850 |
| Theta | Frontal | 8.234 | 0.452 | 0.020 | 0.064 |
|  | Central | 31.307 | 0.758 | 0.005 | 0.026 |
|  | Temporal | 24.810 | 0.713 | 0.002 | 0.018 |
|  | Parietal | 62.873 | 0.863 | 0.002 | 0.018 |
|  | Occipital | 76.238 | 0.884 | 0.002 | 0.024 |
| Alpha | Frontal | 0.054 | 0.005 | 0.854 | 0.856 |
|  | Central | 0.327 | 0.032 | 0.571 | 0.721 |
|  | Temporal | 1.594 | 0.137 | 0.226 | 0.372 |
|  | Parietal | 1.799 | 0.152 | 0.202 | 0.352 |
|  | Occipital | 1.418 | 0.124 | 0.261 | 0.406 |
| Beta | Frontal | 3.772 | 0.274 | 0.098 | 0.188 |
|  | Central | 4.735 | 0.321 | 0.025 | 0.064 |
|  | Temporal | 0.438 | 0.042 | 0.525 | 0.701 |
|  | Parietal | 0.049 | 0.005 | 0.829 | 0.856 |
|  | Occipital | 1.013 | 0.092 | 0.333 | 0.498 |
| Low-Gamma | Frontal | 9.448 | 0.486 | 0.003 | 0.018 |
|  | Central | 4.263 | 0.299 | 0.074 | 0.157 |
|  | Temporal | 4.760 | 0.322 | 0.028 | 0.066 |
|  | Parietal | 3.654 | 0.268 | 0.101 | 0.202 |
|  | Occipital | 7.674 | 0.434 | 0.026 | 0.064 |
| **Supplementary Table S14** PERMANOVA results comparing Sleep Paralysis and REM sleep across frequency bands and brain regions for Subject 4. FDR-adjusted p-values (Benjamini-Hochberg method) are reported. | | | | | |

| **Supplementary Table S15** Spectral power differences between Sleep Paralysis and S1 for Subject 4. | | | | | |
| --- | --- | --- | --- | --- | --- |
| Frequency Band | Brain Region | F-value | R² | p-value | Adjusted p-value |
| Delta | Frontal | 0.017 | 0.002 | 0.898 | 0.967 |
|  | Central | 17.994 | 0.643 | 0.004 | 0.020 |
|  | Temporal | 0.009 | 0.001 | 0.929 | 0.967 |
|  | Parietal | 7.960 | 0.443 | 0.013 | 0.037 |
|  | Occipital | 0.073 | 0.007 | 0.792 | 0.947 |
| Theta | Frontal | 11.225 | 0.529 | 0.004 | 0.020 |
|  | Central | 12.221 | 0.550 | 0.005 | 0.022 |
|  | Temporal | 15.416 | 0.607 | 0.002 | 0.017 |
|  | Parietal | 1.185 | 0.106 | 0.301 | 0.446 |
|  | Occipital | 42.366 | 0.809 | 0.003 | 0.017 |
| Alpha | Frontal | 7.661 | 0.434 | 0.018 | 0.037 |
|  | Central | 0.152 | 0.015 | 0.695 | 0.911 |
|  | Temporal | 1.523 | 0.132 | 0.233 | 0.386 |
|  | Parietal | 15.465 | 0.607 | 0.007 | 0.022 |
|  | Occipital | 1.532 | 0.133 | 0.243 | 0.387 |
| Beta | Frontal | 0.013 | 0.001 | 0.906 | 0.967 |
|  | Central | 11.549 | 0.536 | 0.002 | 0.017 |
|  | Temporal | 0.077 | 0.008 | 0.792 | 0.947 |
|  | Parietal | 6.412 | 0.391 | 0.028 | 0.065 |
|  | Occipital | 0.462 | 0.044 | 0.510 | 0.692 |
| Low-Gamma | Frontal | 7.300 | 0.422 | 0.011 | 0.029 |
|  | Central | 0.000 | 0.000 | 0.994 | 0.994 |
|  | Temporal | 4.060 | 0.289 | 0.045 | 0.086 |
|  | Parietal | 10.727 | 0.518 | 0.002 | 0.017 |
|  | Occipital | 6.839 | 0.406 | 0.049 | 0.086 |
| **Supplementary Table S15** PERMANOVA results comparing Sleep Paralysis and S1 across frequency bands and brain regions for Subject 4. FDR-adjusted p-values (Benjamini-Hochberg method) are reported. | | | | | |

| **Supplementary Table S16** Spectral power differences between Sleep Paralysis and Wakefulness for Subject 4. | | | | | |
| --- | --- | --- | --- | --- | --- |
| Frequency Band | Brain Region | F-value | R² | p-value | Adjusted p-value |
| Delta | Frontal | 3.010 | 0.231 | 0.110 | 0.157 |
|  | Central | 150.590 | 0.938 | 0.003 | 0.010 |
|  | Temporal | 11.350 | 0.532 | 0.013 | 0.031 |
|  | Parietal | 9.255 | 0.481 | 0.019 | 0.034 |
|  | Occipital | 10.917 | 0.522 | 0.002 | 0.010 |
| Theta | Frontal | 0.267 | 0.026 | 0.619 | 0.740 |
|  | Central | 0.093 | 0.009 | 0.774 | 0.858 |
|  | Temporal | 3.273 | 0.247 | 0.095 | 0.144 |
|  | Parietal | 22.604 | 0.693 | 0.002 | 0.010 |
|  | Occipital | 8.105 | 0.448 | 0.008 | 0.018 |
| Alpha | Frontal | 15.687 | 0.611 | 0.009 | 0.025 |
|  | Central | 149.399 | 0.937 | 0.002 | 0.010 |
|  | Temporal | 19.814 | 0.665 | 0.003 | 0.010 |
|  | Parietal | 25.221 | 0.716 | 0.002 | 0.010 |
|  | Occipital | 81.690 | 0.891 | 0.002 | 0.010 |
| Beta | Frontal | 0.001 | 0.000 | 0.979 | 0.979 |
|  | Central | 89.334 | 0.899 | 0.002 | 0.010 |
|  | Temporal | 0.084 | 0.008 | 0.783 | 0.858 |
|  | Parietal | 1.352 | 0.119 | 0.276 | 0.336 |
|  | Occipital | 3.712 | 0.271 | 0.101 | 0.144 |
| Low-Gamma | Frontal | 1.331 | 0.117 | 0.252 | 0.313 |
|  | Central | 3.303 | 0.248 | 0.063 | 0.111 |
|  | Temporal | 0.004 | 0.000 | 0.953 | 0.979 |
|  | Parietal | 5.916 | 0.372 | 0.046 | 0.083 |
|  | Occipital | 6.009 | 0.375 | 0.037 | 0.063 |
| **Supplementary Table S16** PERMANOVA results comparing Sleep Paralysis and Wakefulness across frequency bands and brain regions for Subject 4. FDR-adjusted p-values (Benjamini-Hochberg method) are reported. | | | | | |

**Subject 5**

| **Supplementary Table S17** Subject 5 – Post hoc results comparing sleep states. | | | | | | |
| --- | --- | --- | --- | --- | --- | --- |
|  | Df | Sum of Squares | F-value | R**²** | p-value | Adjusted p-value |
| SP vs REM sleep | 1 | 0.151 | 17.869 | 0.641 | 0.003 | 0.016 |
| SP vs S1 | 1 | 0.067 | 5.894 | 0.371 | 0.005 | 0.032 |
| SP vs Wakefulness | 1 | 0.112 | 11.123 | 0.527 | 0.002 | 0.013 |
| REM sleep vs Wakefulness | 1 | 0.520 | 78.842 | 0.887 | 0.002 | 0.014 |
| REM sleep vs S1 | 1 | 0.033 | 4.250 | 0.298 | 0.005 | 0.031 |
| S1 vs Wakefulness | 1 | 0.342 | 36.220 | 0.784 | 0.003 | 0.016 |
| **Supplementary Table S17** Post hoc PERMANOVA results comparing Sleep Paralysis, REM sleep, S1, and Wakefulness for Subject 5. Bonferroni-adjusted p-values are reported. | | | | | | |

| **Supplementary Table S18** Spectral power differences between Sleep Paralysis and REM sleep for Subject 5. | | | | | |
| --- | --- | --- | --- | --- | --- |
| Frequency Band | Brain Region | F-value | R² | p-value | Adjusted p-value |
| Delta | Frontal | 19.763 | 0.664 | 0.002 | 0.010 |
|  | Central | 38.168 | 0.792 | 0.002 | 0.010 |
|  | Temporal | 19.102 | 0.656 | 0.004 | 0.012 |
|  | Parietal | 23.797 | 0.704 | 0.002 | 0.010 |
|  | Occipital | 21.346 | 0.681 | 0.006 | 0.013 |
| Theta | Frontal | 2.621 | 0.208 | 0.130 | 0.132 |
|  | Central | 4.018 | 0.287 | 0.082 | 0.086 |
|  | Temporal | 12.590 | 0.557 | 0.008 | 0.018 |
|  | Parietal | 14.079 | 0.585 | 0.004 | 0.012 |
|  | Occipital | 14.920 | 0.599 | 0.011 | 0.018 |
| Alpha | Frontal | 16.836 | 0.627 | 0.008 | 0.018 |
|  | Central | 9.813 | 0.495 | 0.016 | 0.020 |
|  | Temporal | 9.092 | 0.476 | 0.011 | 0.018 |
|  | Parietal | 10.744 | 0.518 | 0.014 | 0.020 |
|  | Occipital | 7.664 | 0.434 | 0.016 | 0.020 |
| Beta | Frontal | 22.775 | 0.695 | 0.002 | 0.010 |
|  | Central | 12.645 | 0.558 | 0.005 | 0.012 |
|  | Temporal | 18.241 | 0.646 | 0.002 | 0.010 |
|  | Parietal | 13.471 | 0.574 | 0.002 | 0.010 |
|  | Occipital | 15.121 | 0.602 | 0.002 | 0.010 |
| Low-Gamma | Frontal | 11.859 | 0.543 | 0.012 | 0.018 |
|  | Central | 8.881 | 0.470 | 0.018 | 0.020 |
|  | Temporal | 5.139 | 0.339 | 0.043 | 0.048 |
|  | Parietal | 7.670 | 0.434 | 0.017 | 0.021 |
|  | Occipital | 3.459 | 0.257 | 0.090 | 0.093 |
| **Supplementary Table S18** PERMANOVA results comparing Sleep Paralysis and REM sleep across frequency bands and brain regions for Subject 5. FDR-adjusted p-values (Benjamini-Hochberg method) are reported. | | | | | |

| **Supplementary Table S19** Spectral power differences between Sleep Paralysis and S1 for Subject 5. | | | | | |
| --- | --- | --- | --- | --- | --- |
| Frequency Band | Brain Region | F-value | R² | p-value | Adjusted p-value |
| Delta | Frontal | 1.069 | 0.097 | 0.329 | 0.388 |
|  | Central | 7.864 | 0.440 | 0.027 | 0.049 |
|  | Temporal | 0.231 | 0.023 | 0.626 | 0.656 |
|  | Parietal | 0.944 | 0.086 | 0.325 | 0.388 |
|  | Occipital | 2.953 | 0.228 | 0.114 | 0.179 |
| Theta | Frontal | 9.607 | 0.490 | 0.013 | 0.037 |
|  | Central | 10.309 | 0.508 | 0.002 | 0.028 |
|  | Temporal | 13.929 | 0.582 | 0.006 | 0.028 |
|  | Parietal | 13.033 | 0.566 | 0.003 | 0.028 |
|  | Occipital | 8.951 | 0.472 | 0.015 | 0.036 |
| Alpha | Frontal | 4.863 | 0.327 | 0.056 | 0.112 |
|  | Central | 3.292 | 0.248 | 0.078 | 0.134 |
|  | Temporal | 0.325 | 0.031 | 0.588 | 0.650 |
|  | Parietal | 0.937 | 0.086 | 0.364 | 0.405 |
|  | Occipital | 1.063 | 0.096 | 0.312 | 0.388 |
| Beta | Frontal | 8.909 | 0.471 | 0.012 | 0.036 |
|  | Central | 7.852 | 0.440 | 0.005 | 0.028 |
|  | Temporal | 6.396 | 0.390 | 0.017 | 0.036 |
|  | Parietal | 6.588 | 0.397 | 0.014 | 0.036 |
|  | Occipital | 9.865 | 0.497 | 0.004 | 0.028 |
| Low-Gamma | Frontal | 2.566 | 0.204 | 0.147 | 0.205 |
|  | Central | 15.302 | 0.605 | 0.004 | 0.028 |
|  | Temporal | 0.077 | 0.008 | 0.778 | 0.774 |
|  | Parietal | 6.408 | 0.391 | 0.034 | 0.063 |
|  | Occipital | 1.862 | 0.157 | 0.209 | 0.286 |
| **Supplementary Table S19** PERMANOVA results comparing Sleep Paralysis and S1 across frequency bands and brain regions for Subject 5. FDR-adjusted p-values (Benjamini-Hochberg method) are reported. | | | | | |

| **Supplementary Table S20** Spectral power differences between Sleep Paralysis and Wakefulness for Subject 5. | | | | | |
| --- | --- | --- | --- | --- | --- |
| Frequency Band | Brain Region | F-value | R² | p-value | Adjusted p-value |
| Delta | Frontal | 1.338 | 0.118 | 0.301 | 0.355 |
|  | Central | 9.264 | 0.481 | 0.027 | 0.049 |
|  | Temporal | 62.499 | 0.862 | 0.002 | 0.013 |
|  | Parietal | 12.269 | 0.551 | 0.011 | 0.024 |
|  | Occipital | 4.975 | 0.332 | 0.044 | 0.075 |
| Theta | Frontal | 14.582 | 0.593 | 0.010 | 0.024 |
|  | Central | 13.206 | 0.569 | 0.008 | 0.024 |
|  | Temporal | 31.190 | 0.757 | 0.001 | 0.013 |
|  | Parietal | 11.629 | 0.538 | 0.007 | 0.024 |
|  | Occipital | 11.377 | 0.532 | 0.002 | 0.013 |
| Alpha | Frontal | 0.064 | 0.006 | 0.807 | 0.808 |
|  | Central | 2.295 | 0.187 | 0.155 | 0.207 |
|  | Temporal | 0.088 | 0.009 | 0.769 | 0.803 |
|  | Parietal | 4.356 | 0.303 | 0.048 | 0.079 |
|  | Occipital | 2.386 | 0.193 | 0.143 | 0.192 |
| Beta | Frontal | 0.909 | 0.083 | 0.344 | 0.371 |
|  | Central | 1.179 | 0.105 | 0.312 | 0.355 |
|  | Temporal | 10.280 | 0.507 | 0.015 | 0.039 |
|  | Parietal | 2.544 | 0.203 | 0.131 | 0.192 |
|  | Occipital | 1.403 | 0.123 | 0.254 | 0.321 |
| Low-Gamma | Frontal | 10.683 | 0.517 | 0.012 | 0.024 |
|  | Central | 12.208 | 0.550 | 0.005 | 0.020 |
|  | Temporal | 57.528 | 0.852 | 0.002 | 0.013 |
|  | Parietal | 6.247 | 0.385 | 0.029 | 0.050 |
|  | Occipital | 5.812 | 0.368 | 0.029 | 0.050 |
| **Supplementary Table S20** PERMANOVA results comparing Sleep Paralysis and Wakefulness across frequency bands and brain regions for Subject 5. FDR-adjusted p-values (Benjamini-Hochberg method) are reported. | | | | | |

**Out-of-body Experiences**

**PCA Results**

**Subject 3 – OBE_1_**

| **Supplementary Table S21** Subject 3 (OBE_1_) – Principal Component Analysis: Contribution and Representation of Conditions | | | | |
| --- | --- | --- | --- | --- |
| Condition | % Contribution PC 1 | % Contribution PC 2 | Cos² PC 1 | Cos² PC 2 |
| OBE_1_ | 20.363 | 16.331 | 0.615 | 0.126 |
| REM sleep | 5.354 | 25.679 | 0.372 | 0.232 |
| S1 | 8.120 | 44.592 | 0.25 | 0.417 |
| Wakefulness | 66.164 | 13.398 | 0.829 | 0.05 |
| **Supplementary Table S21** Principal Component Analysis results for Subject 3 (OBE_1_). The table presents the percentage of contribution and squared cosine (cos²) values for each condition across the first two principal components (PC1 and PC2). | | | | |

**
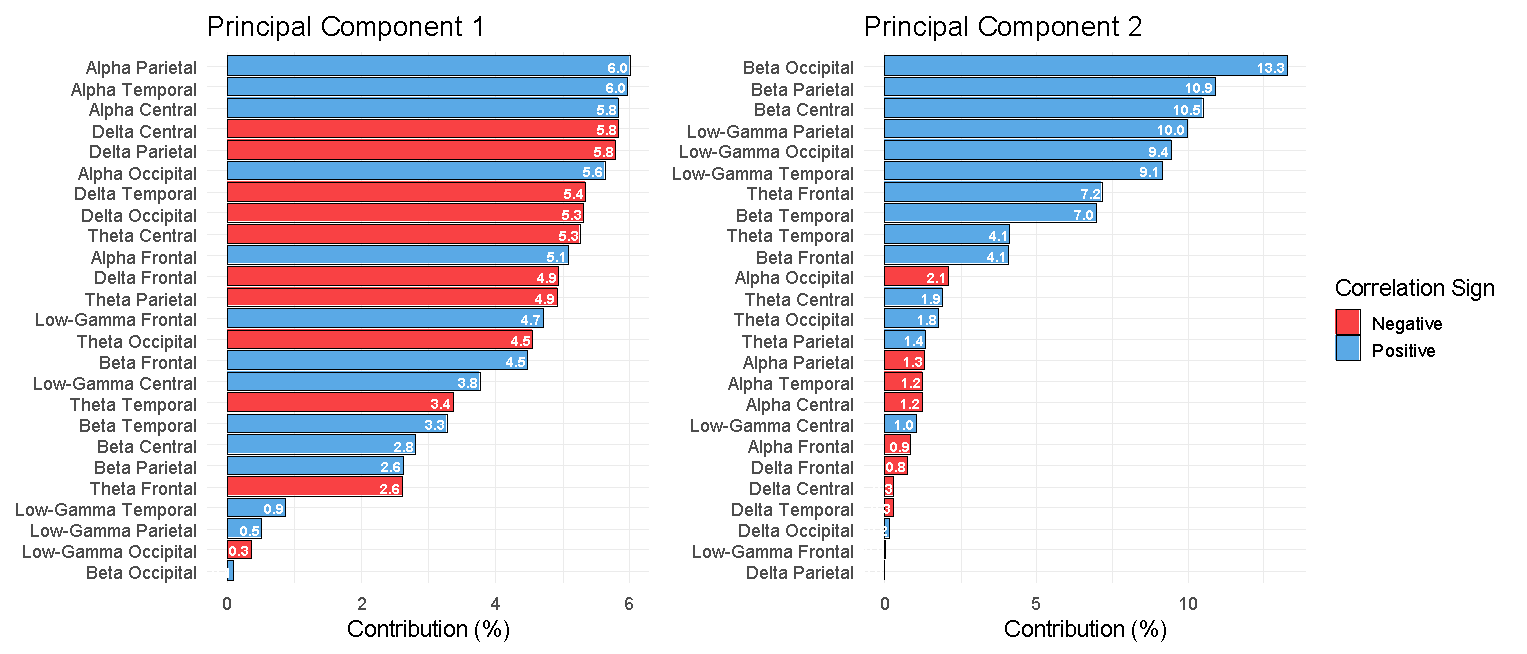
**

**Supplementary Figure S6** Contributions of EEG spectral variables to the first two principal components in Subject 3 – OBE_1_. Each bar represents a variable defined by a frequency band and cortical region, with bar length indicating its percentage contribution to the corresponding component. Colors represent the sign of the correlation between the variable and the component: blue for positive and red for negative. Variables are ordered by contribution within each component.

**Subject 3 – OBE_2_**

| **Supplementary Table S22** Subject 3 (OBE_2_) – Principal Component Analysis: Contribution and Representation of Conditions | | | | |
| --- | --- | --- | --- | --- |
| Condition | % Contribution PC 1 | % Contribution PC 2 | Cos² PC 1 | Cos² PC 2 |
| OBE_2_ | 15.820 | 15.265 | 0.398 | 0.353 |
| REM sleep | 16.580 | 7.643 | 0.542 | 0.158 |
| S1 | 4.178 | 69.770 | 0.120 | 0.679 |
| Wakefulness | 63.423 | 7.322 | 0.889 | 0.068 |
| **Supplementary Table S22** Principal Component Analysis results for Subject 3 (OBE_2_). The table presents the percentage of contribution and squared cosine (cos²) values for each condition across the first two principal components (PC1 and PC2). | | | | |

*
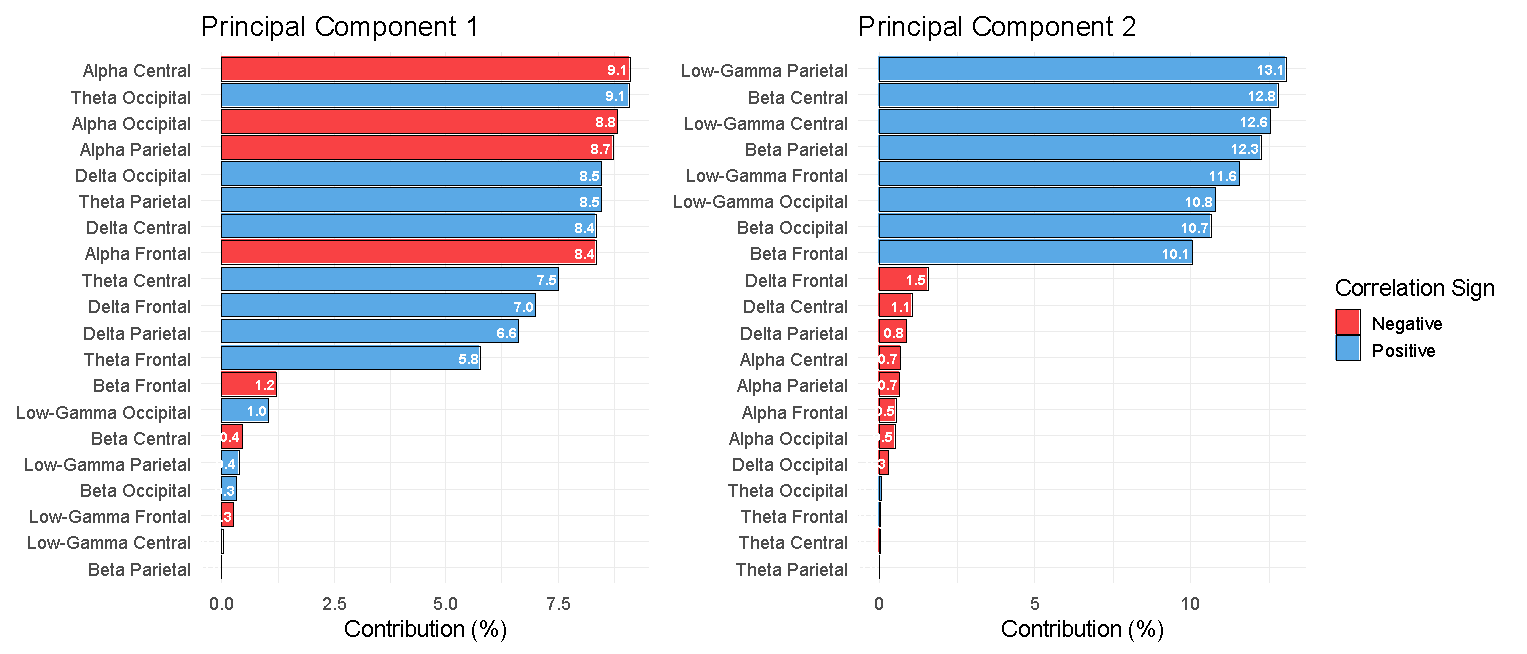
*

**Supplementary Figure S7** Contributions of EEG spectral variables to the first two principal components in Subject 3 – OBE_2_. Each bar represents a variable defined by a frequency band and cortical region, with bar length indicating its percentage contribution to the corresponding component. Colors represent the sign of the correlation between the variable and the component: blue for positive and red for negative. Variables are ordered by contribution within each component.

**PERMANOVA Results**

**Subject 3 - OBE_1_**

| **Supplementary Table S23** Subject 3 (OBE_1_) – Post hoc results comparing sleep states. | | | | | | |
| --- | --- | --- | --- | --- | --- | --- |
| Comparison | Df | Sum Of Squares | F-value | R² | p-value | Adjusted p-value |
| OBE_1_ vs REM sleep | 1 | 0.051 | 6.075 | 0.378 | 0.008 | 0.049 |
| OBE_1_ vs S1 | 1 | 0.102 | 11.652 | 0.538 | 0.005 | 0.029 |
| OBE_1_ vs Wakefulness | 1 | 1.170 | 178.139 | 0.947 | 0.002 | 0.010 |
| REM sleep vs Wakefulness | 1 | 0.830 | 110.759 | 0.917 | 0.001 | 0.008 |
| S1 vs REM sleep | 1 | 0.015 | 1.600 | 0.138 | 0.157 | 0.944 |
| S1 vs Wakefulness | 1 | 0.849 | 108.134 | 0.915 | 0.002 | 0.014 |
| **Supplementary Table S23** Post hoc PERMANOVA results comparing OBE_1_, REM sleep, S1, and Wakefulness for Subject 3. Bonferroni-adjusted p-values are reported. | | | | | | |

| **Supplementary Table S24** Spectral power differences between OBE_1_ and REM sleep for Subject 3. | | | | | |
| --- | --- | --- | --- | --- | --- |
| Frequency Band | Brain Region | F-value | R² | p-value | Adjusted p-value |
| Delta | Frontal | 7.618 | 0.432 | 0.014 | 0.042 |
|  | Central | 4.544 | 0.312 | 0.057 | 0.113 |
|  | Temporal | 23.155 | 0.698 | 0.004 | 0.029 |
|  | Parietal | 8.304 | 0.454 | 0.020 | 0.061 |
|  | Occipital | 0.133 | 0.013 | 0.715 | 0.744 |
| Theta | Frontal | 2.409 | 0.194 | 0.157 | 0.244 |
|  | Central | 0.201 | 0.020 | 0.649 | 0.698 |
|  | Temporal | 3.057 | 0.234 | 0.114 | 0.169 |
|  | Parietal | 1.019 | 0.093 | 0.342 | 0.419 |
|  | Occipital | 0.422 | 0.040 | 0.548 | 0.648 |
| Alpha | Frontal | 13.203 | 0.569 | 0.008 | 0.029 |
|  | Central | 5.636 | 0.360 | 0.044 | 0.101 |
|  | Temporal | 19.346 | 0.659 | 0.004 | 0.029 |
|  | Parietal | 6.936 | 0.410 | 0.025 | 0.061 |
|  | Occipital | 0.381 | 0.037 | 0.586 | 0.671 |
| Beta | Frontal | 12.239 | 0.550 | 0.005 | 0.029 |
|  | Central | 12.580 | 0.557 | 0.005 | 0.029 |
|  | Temporal | 24.777 | 0.712 | 0.002 | 0.029 |
|  | Parietal | 4.170 | 0.294 | 0.066 | 0.113 |
|  | Occipital | 1.573 | 0.136 | 0.235 | 0.322 |
| Low-Gamma | Frontal | 4.559 | 0.313 | 0.067 | 0.113 |
|  | Central | 3.911 | 0.281 | 0.067 | 0.113 |
|  | Temporal | 4.992 | 0.333 | 0.044 | 0.101 |
|  | Parietal | 1.176 | 0.105 | 0.305 | 0.406 |
|  | Occipital | 0.027 | 0.003 | 0.878 | 0.877 |
| **Supplementary Table S24** PERMANOVA results comparing OBE_1_ and REM sleep across frequency bands and brain regions for Subject 3. FDR-adjusted p-values (Benjamini-Hochberg method) are reported. | | | | | |

| **Supplementary Table S25** Spectral power differences between OBE_1_ and S1 for Subject 3. | | | | | |
| --- | --- | --- | --- | --- | --- |
| Frequency Band | Brain Region | F-value | R² | p-value | Adjusted p-value |
| Delta | Frontal | 36.669 | 0.786 | 0.002 | 0.017 |
|  | Central | 10.002 | 0.500 | 0.013 | 0.039 |
|  | Temporal | 45.653 | 0.820 | 0.002 | 0.017 |
|  | Parietal | 12.719 | 0.560 | 0.008 | 0.026 |
|  | Occipital | 1.736 | 0.148 | 0.218 | 0.279 |
| Theta | Frontal | 22.606 | 0.693 | 0.006 | 0.020 |
|  | Central | 0.054 | 0.005 | 0.831 | 0.862 |
|  | Temporal | 18.038 | 0.643 | 0.005 | 0.020 |
|  | Parietal | 3.173 | 0.241 | 0.108 | 0.163 |
|  | Occipital | 1.590 | 0.137 | 0.239 | 0.284 |
| Alpha | Frontal | 11.775 | 0.541 | 0.011 | 0.027 |
|  | Central | 4.096 | 0.291 | 0.073 | 0.132 |
|  | Temporal | 13.415 | 0.573 | 0.012 | 0.027 |
|  | Parietal | 3.247 | 0.245 | 0.104 | 0.163 |
|  | Occipital | 0.391 | 0.038 | 0.541 | 0.603 |
| Beta | Frontal | 12.430 | 0.554 | 0.010 | 0.027 |
|  | Central | 10.909 | 0.522 | 0.009 | 0.027 |
|  | Temporal | 15.369 | 0.606 | 0.003 | 0.017 |
|  | Parietal | 4.832 | 0.326 | 0.056 | 0.098 |
|  | Occipital | 3.404 | 0.254 | 0.091 | 0.156 |
| Low-Gamma | Frontal | 8.119 | 0.448 | 0.019 | 0.040 |
|  | Central | 2.522 | 0.201 | 0.138 | 0.187 |
|  | Temporal | 1.541 | 0.134 | 0.240 | 0.284 |
|  | Parietal | 0.327 | 0.032 | 0.577 | 0.628 |
|  | Occipital | 0.016 | 0.002 | 0.912 | 0.913 |
| **Supplementary Table S25** PERMANOVA results comparing OBE_1_ and S1 across frequency bands and brain regions for Subject 3. FDR-adjusted p-values (Benjamini-Hochberg method) are reported. | | | | | |

| **Supplementary Table S26** Spectral power differences between OBE_1_ and Wakefulness for Subject 3. | | | | | |
| --- | --- | --- | --- | --- | --- |
| Frequency Band | Brain Region | F-value | R² | p-value | Adjusted p-value |
| Delta | Frontal | 71.637 | 0.878 | 0.002 | 0.003 |
|  | Central | 113.796 | 0.919 | 0.002 | 0.003 |
|  | Temporal | 223.682 | 0.957 | 0.003 | 0.003 |
|  | Parietal | 136.905 | 0.932 | 0.002 | 0.003 |
|  | Occipital | 59.562 | 0.856 | 0.002 | 0.003 |
| Theta | Frontal | 23.468 | 0.701 | 0.002 | 0.003 |
|  | Central | 195.762 | 0.951 | 0.003 | 0.003 |
|  | Temporal | 24.970 | 0.714 | 0.002 | 0.003 |
|  | Parietal | 126.904 | 0.927 | 0.002 | 0.003 |
|  | Occipital | 59.251 | 0.856 | 0.002 | 0.003 |
| Alpha | Frontal | 31.757 | 0.761 | 0.002 | 0.003 |
|  | Central | 145.223 | 0.936 | 0.002 | 0.003 |
|  | Temporal | 1.491.177 | 0.993 | 0.002 | 0.003 |
|  | Parietal | 880.691 | 0.989 | 0.002 | 0.003 |
|  | Occipital | 394.371 | 0.975 | 0.002 | 0.003 |
| Beta | Frontal | 107.931 | 0.915 | 0.002 | 0.003 |
|  | Central | 61.049 | 0.859 | 0.002 | 0.003 |
|  | Temporal | 143.015 | 0.935 | 0.003 | 0.003 |
|  | Parietal | 31.544 | 0.759 | 0.003 | 0.006 |
|  | Occipital | 0.576 | 0.054 | 0.459 | 0.473 |
| Low-Gamma | Frontal | 24.852 | 0.713 | 0.003 | 0.003 |
|  | Central | 9.800 | 0.495 | 0.002 | 0.003 |
|  | Temporal | 9.899 | 0.497 | 0.013 | 0.016 |
|  | Parietal | 0.416 | 0.040 | 0.707 | 0.710 |
|  | Occipital | 2.745 | 0.215 | 0.137 | 0.143 |
| **Supplementary Table S26** PERMANOVA results comparing OBE_1_ and Wakefulness across frequency bands and brain regions for Subject 3. FDR-adjusted p-values (Benjamini-Hochberg method) are reported. | | | | | |

**Subject 3 - OBE_2_**

| **Supplementary table S27** Post hoc PERMANOVA results for Subject 3 – OBE_2_. | | | | | | |
| --- | --- | --- | --- | --- | --- | --- |
| Comparison | Df | Sum of Squares | F-value | R² | p-value | Adjusted p-value |
| OBE_2_ vs REM sleep | 1 | 0.020 | 1.524 | 0.132 | 0.202 | 1.000 |
| OBE_2_ vs S1 | 1 | 0.174 | 11.616 | 0.537 | 0.003 | 0.017 |
| OBE_2_ vs Wakefulness | 1 | 0.779 | 74.655 | 0.882 | 0.002 | 0.013 |
| REM vs Wakefulness | 1 | 0.798 | 116.681 | 0.921 | 0.002 | 0.013 |
| S1 vs REM sleep | 1 | 0.136 | 11.935 | 0.544 | 0.004 | 0.023 |
| S1 vs Wakefulness | 1 | 0.574 | 67.746 | 0.871 | 0.002 | 0.012 |
| **Supplementary Table S27** Post hoc PERMANOVA results comparing OBE_2_ and control conditions (REM sleep, S1, and Wakefulness) for Subject 3 OBE_2_. P-values were Bonferroni-adjusted for multiple comparisons.. | | | | | | |

| **Supplementary Table S28** Spectral power differences between OBE_2_ and S1 for Subject 3. | | | | | |
| --- | --- | --- | --- | --- | --- |
| Frequency Band | Brain Region | F-value | R² | p-value | Adjusted p-value |
| Delta | Frontal | 33.756 | 0.771 | 0.002 | 0.007 |
|  | Central | 38.790 | 0.795 | 0.002 | 0.007 |
|  | Parietal | 11.258 | 0.530 | 0.004 | 0.011 |
|  | Occipital | 4.495 | 0.310 | 0.086 | 0.132 |
| Theta | Frontal | 0.320 | 0.031 | 0.622 | 0.652 |
|  | Central | 0.274 | 0.027 | 0.611 | 0.652 |
|  | Parietal | 0.720 | 0.067 | 0.412 | 0.512 |
|  | Occipital | 0.611 | 0.058 | 0.503 | 0.592 |
| Alpha | Frontal | 1.722 | 0.147 | 0.230 | 0.320 |
|  | Central | 1.761 | 0.150 | 0.221 | 0.320 |
|  | Parietal | 1.009 | 0.092 | 0.322 | 0.434 |
|  | Occipital | 0.009 | 0.001 | 0.921 | 0.922 |
| Beta | Frontal | 25.342 | 0.717 | 0.003 | 0.007 |
|  | Central | 39.200 | 0.797 | 0.002 | 0.007 |
|  | Parietal | 24.757 | 0.712 | 0.004 | 0.010 |
|  | Occipital | 27.764 | 0.735 | 0.004 | 0.010 |
| Low-Gamma | Frontal | 23.013 | 0.697 | 0.002 | 0.007 |
|  | Central | 14.016 | 0.584 | 0.003 | 0.007 |
|  | Parietal | 16.520 | 0.623 | 0.003 | 0.010 |
|  | Occipital | 17.558 | 0.637 | 0.002 | 0.007 |
| **Supplementary Table S28.** PERMANOVA results comparing OBE_2_ and S1 across frequency bands in the central region for Subject 3. FDR-adjusted p-values (Benjamini-Hochberg method) are reported. | | | | | |

| **Supplementary Table S29** Spectral power differences between OBE_2_ and Wakefulness for Subject 3. | | | | | |
| --- | --- | --- | --- | --- | --- |
| Frequency Band | Brain Region | F-value | R² | p-value | Adjusted p-value |
| Delta | Frontal | 40.785 | 0.803 | 0.002 | 0.005 |
|  | Central | 124.782 | 0.926 | 0.002 | 0.005 |
|  | Parietal | 33.033 | 0.768 | 0.002 | 0.005 |
|  | Occipital | 28.273 | 0.739 | 0.003 | 0.005 |
| Theta | Frontal | 132.186 | 0.930 | 0.003 | 0.005 |
|  | Central | 66.980 | 0.870 | 0.002 | 0.005 |
|  | Parietal | 343.443 | 0.972 | 0.002 | 0.005 |
|  | Occipital | 74.515 | 0.882 | 0.002 | 0.005 |
| Alpha | Frontal | 51.762 | 0.838 | 0.002 | 0.005 |
|  | Central | 305.430 | 0.968 | 0.002 | 0.005 |
|  | Parietal | 98.602 | 0.908 | 0.002 | 0.005 |
|  | Occipital | 35.530 | 0.780 | 0.002 | 0.005 |
| Beta | Frontal | 10.630 | 0.515 | 0.010 | 0.014 |
|  | Central | 6.784 | 0.404 | 0.032 | 0.038 |
|  | Parietal | 0.325 | 0.031 | 0.634 | 0.628 |
|  | Occipital | 0.351 | 0.034 | 0.607 | 0.628 |
| Low-Gamma | Frontal | 3.022 | 0.232 | 0.068 | 0.072 |
|  | Central | 1.570 | 0.136 | 0.277 | 0.308 |
|  | Parietal | 4.815 | 0.325 | 0.005 | 0.006 |
|  | Occipital | 14.320 | 0.589 | 0.006 | 0.010 |
| **Supplementary Table S29** PERMANOVA results comparing OBE_2_ and Wakefulness across frequency bands in the central region for Subject 3. FDR-adjusted p-values (Benjamini-Hochberg method) are reported. | | | | | |

**False Awakening**

**PCA Results**

**Subject 3**

| **Supplementary Table S30** Subject 3 – Principal Component Analysis: Contribution and Representation of Conditions | | | | |
| --- | --- | --- | --- | --- |
| Condition | % Contribution PC 1 | % Contribution PC 2 | Cos² PC 1 | Cos² PC 2 |
| FA | 11.971 | 35.685 | 0.235 | 0.322 |
| REM sleep | 34.473 | 34.063 | 0.68 | 0.169 |
| S1 | 1.854 | 17.299 | 0.131 | 0.315 |
| Wakefulness | 51.702 | 12.953 | 0.732 | 0.055 |
| **Supplementary Table S30** Principal Component Analysis results for Subject 3. The table presents the percentage of contribution and squared cosine (cos²) values for each condition across the first two principal components (PC1 and PC2). | | | | |

*
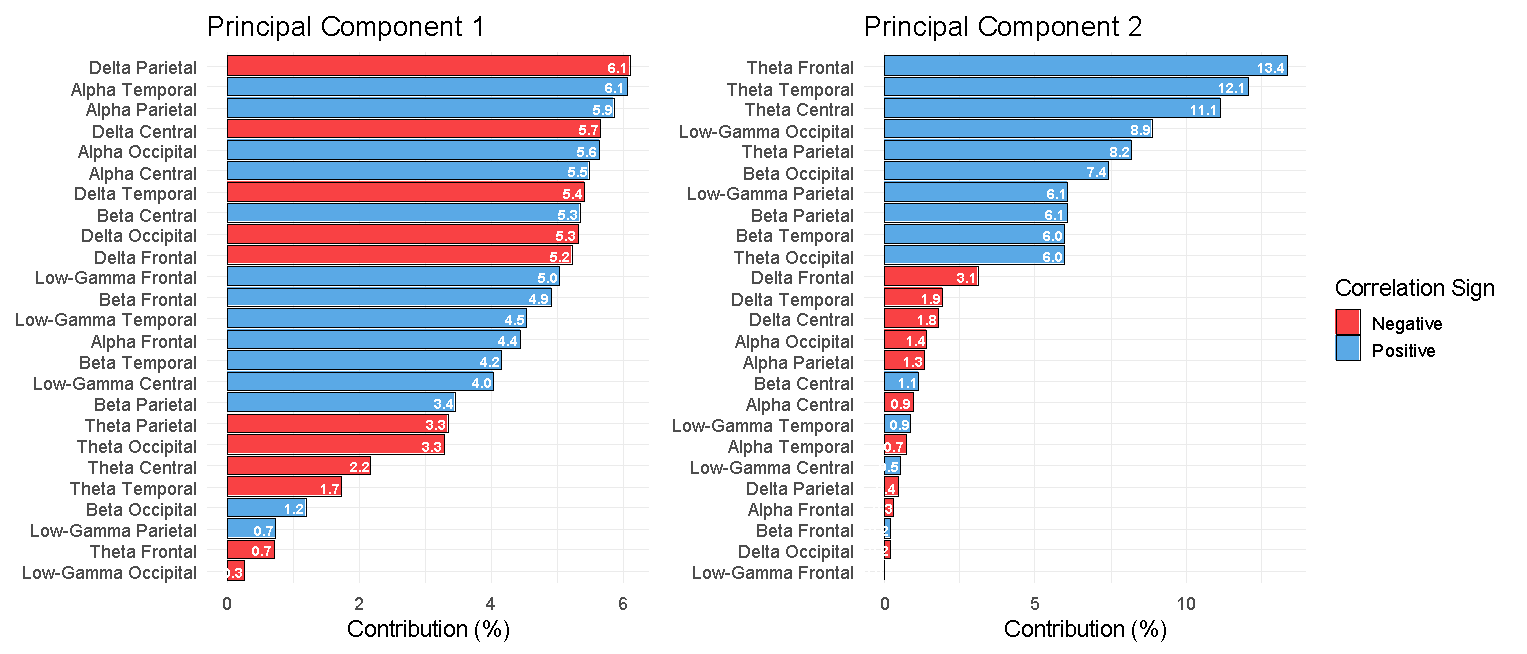
*

**Supplementary Figure S8** Contributions of EEG spectral variables to the first two principal components in Subject 3. Each bar represents a variable defined by a frequency band and cortical region, with bar length indicating its percentage contribution to the corresponding component. Colors represent the sign of the correlation between the variable and the component: blue for positive and red for negative. Variables are ordered by contribution within each component.

**Subject 6**

| **Supplementary Table S31** Subject 6 – Principal Component Analysis: Contribution and Representation of Conditions | | | | |
| --- | --- | --- | --- | --- |
| Condition | % Contribution PC 1 | % Contribution PC 2 | Cos² PC 1 | Cos² PC 2 |
| FA | 15.820 | 15.265 | 0.398 | 0.353 |
| REM sleep | 16.580 | 7.643 | 0.542 | 0.158 |
| S1 | 4.178 | 69.770 | 0.12 | 0.679 |
| Wakefulness | 63.423 | 7.322 | 0.889 | 0.068 |
| **Supplementary Table S31** Principal Component Analysis results for Subject 6. The table presents the percentage of contribution and squared cosine (cos²) values for each condition across the first two principal components (PC1 and PC2). | | | | |

*
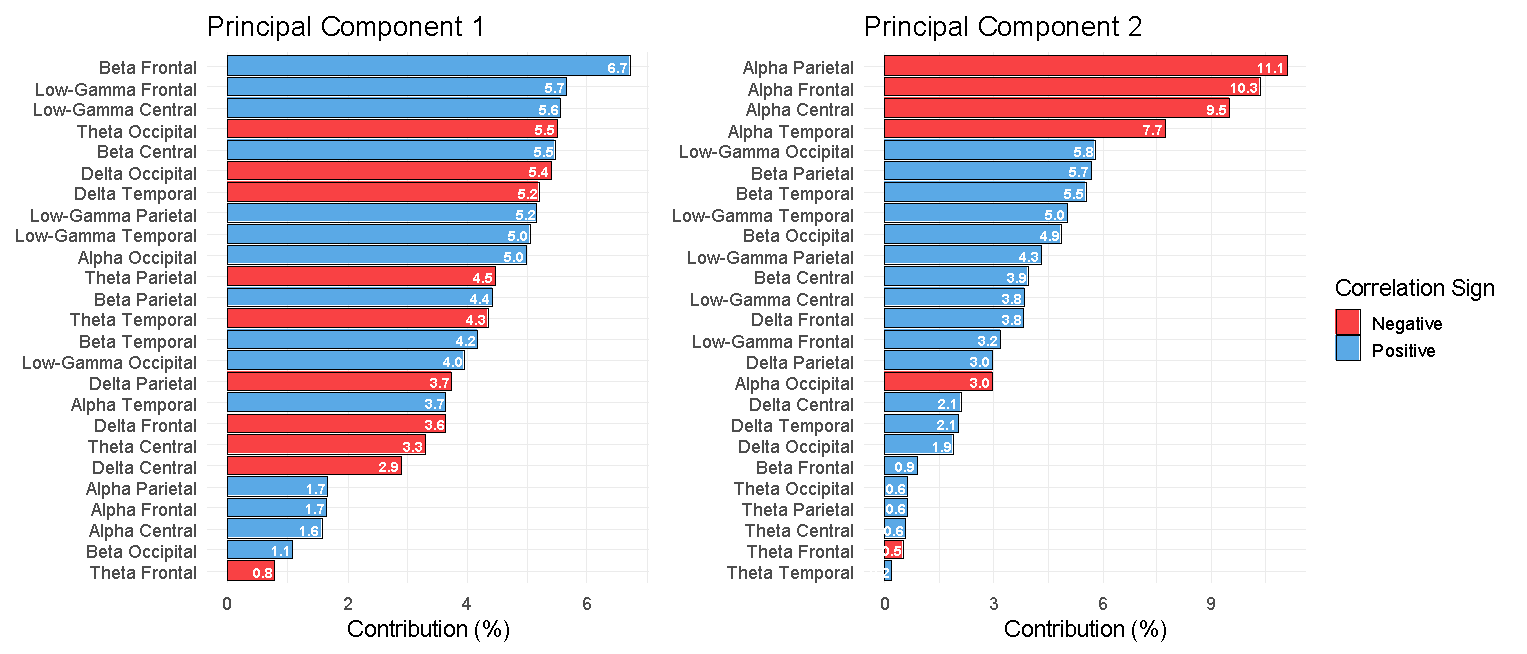
*

**Supplementary Figure S9** Contributions of EEG spectral variables to the first two principal components in Subject 6. Each bar represents a variable defined by a frequency band and cortical region, with bar length indicating its percentage contribution to the corresponding component. Colors represent the sign of the correlation between the variable and the component: blue for positive and red for negative. Variables are ordered by contribution within each component.

**Subject 7**

| **Supplementary Table S32** Subject 7 – Principal Component Analysis: Contribution and Representation of Conditions | | | | |
| --- | --- | --- | --- | --- |
| Condition | % Contribution PC 1 | % Contribution PC 2 | Cos² PC 1 | Cos² PC 2 |
| FA | 12.588 | 16.375 | 0.224 | 0.578 |
| REM sleep | 24.021 | 0.638 | 0.773 | 0.028 |
| S1 | 20.528 | 48.345 | 0.381 | 0.449 |
| Wakefulness | 42.864 | 34.642 | 0.7 | 0.271 |
| **Supplementary Table S32** Principal Component Analysis results for Subject 7. The table presents the percentage of contribution and squared cosine (cos²) values for each condition across the first two principal components (PC1 and PC2). | | | | |


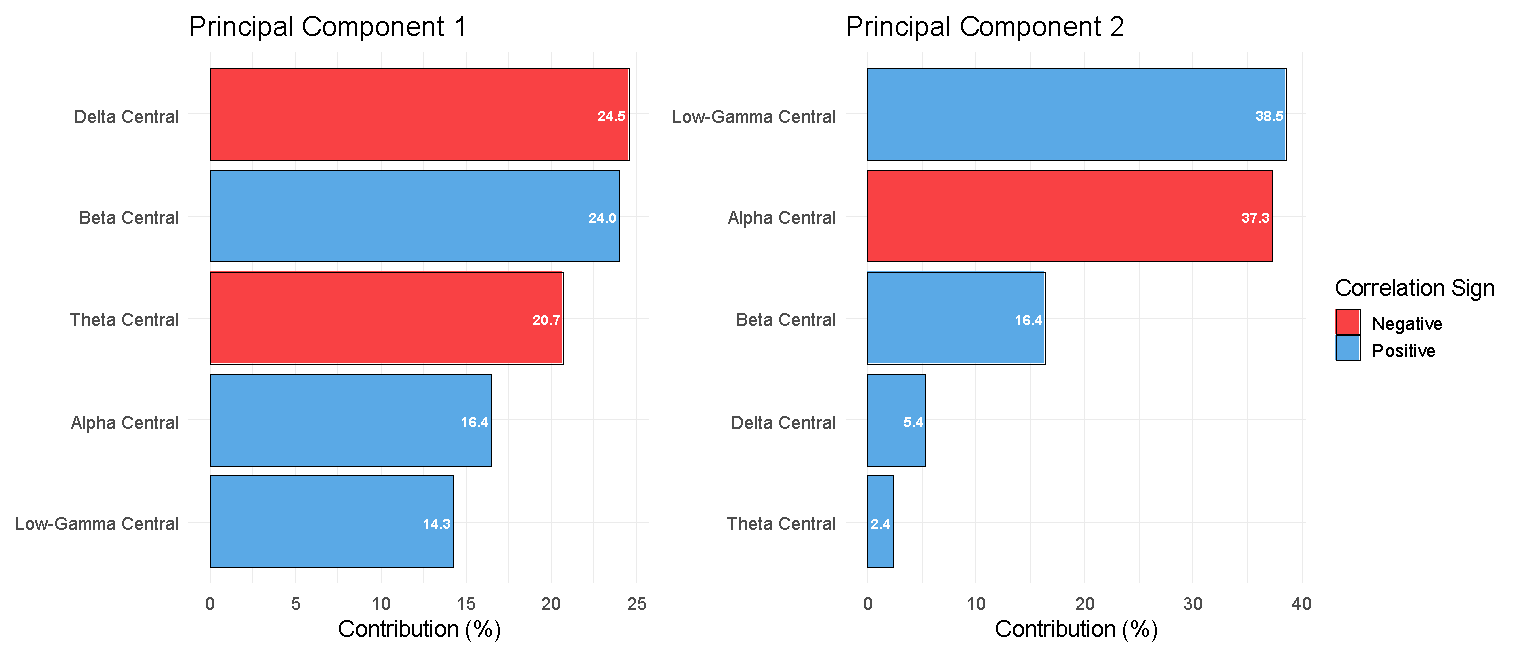


**Supplementary Figure S10** Contributions of EEG spectral variables to the first two principal components in Subject 7. Each bar represents a variable defined by a frequency band and cortical region, with bar length indicating its percentage contribution to the corresponding component. Colors represent the sign of the correlation between the variable and the component: blue for positive and red for negative. Variables are ordered by contribution within each component.

**PERMANOVA Results**

**Subject 3**

| **Supplementary Table S33** Subject 3 Post hoc results comparing sleep states. | | | | | | |
| --- | --- | --- | --- | --- | --- | --- |
| Comparison | Df | Sums Of Squares | F-value | R² | p-value | Adjusted p-value |
| False Awakening vs REM sleep | 1 | 0.172 | 9.252 | 0.481 | 0.005 | 0.031 |
| False Awakening vs S1 | 1 | 0.015 | 0.780 | 0.072 | 0.545 | 1.000 |
| False Awakening vs Wakefulness | 1 | 0.326 | 15.087 | 0.601 | 0.004 | 0.023 |
| REM sleep vs Wakefulness | 1 | 0.765 | 67.174 | 0.870 | 0.002 | 0.011 |
| S1 vs REM sleep | 1 | 0.139 | 15.382 | 0.606 | 0.002 | 0.010 |
| S1 vs Wakefulness | 1 | 0.304 | 25.153 | 0.716 | 0.002 | 0.013 |
| **Supplementary Table S33.** Post hoc PERMANOVA results comparing False Awakening and control conditions (REM sleep, S1, and Wakefulness) for Subject 3. P-values were Bonferroni-adjusted for multiple comparisons.. | | | | | | |

| **Supplementary Table S34** Spectral power differences between False Awakening and REM sleep for Subject 3. | | | | | |
| --- | --- | --- | --- | --- | --- |
| Frequency Band | Brain Region | F-value | R² | p-value | Adjusted p-value |
| Delta | Frontal | 31.740 | 0.760 | 0.002 | 0.027 |
|  | Central | 17.302 | 0.634 | 0.007 | 0.027 |
|  | Temporal | 21.796 | 0.685 | 0.005 | 0.027 |
|  | Parietal | 17.693 | 0.639 | 0.002 | 0.027 |
|  | Occipital | 12.647 | 0.558 | 0.006 | 0.033 |
| Theta | Frontal | 12.448 | 0.555 | 0.010 | 0.033 |
|  | Central | 3.356 | 0.251 | 0.096 | 0.152 |
|  | Temporal | 7.215 | 0.419 | 0.022 | 0.053 |
|  | Parietal | 1.480 | 0.129 | 0.272 | 0.332 |
|  | Occipital | 1.344 | 0.119 | 0.286 | 0.336 |
| Alpha | Frontal | 9.137 | 0.477 | 0.018 | 0.040 |
|  | Central | 3.329 | 0.250 | 0.032 | 0.057 |
|  | Temporal | 3.498 | 0.259 | 0.040 | 0.072 |
|  | Parietal | 3.289 | 0.248 | 0.059 | 0.104 |
|  | Occipital | 2.675 | 0.211 | 0.138 | 0.181 |
| Beta | Frontal | 6.734 | 0.402 | 0.012 | 0.033 |
|  | Central | 9.356 | 0.483 | 0.017 | 0.039 |
|  | Temporal | 23.556 | 0.702 | 0.004 | 0.027 |
|  | Parietal | 7.725 | 0.436 | 0.025 | 0.056 |
|  | Occipital | 2.984 | 0.230 | 0.115 | 0.168 |
| Low-Gamma | Frontal | 2.325 | 0.189 | 0.123 | 0.168 |
|  | Central | 0.835 | 0.077 | 0.379 | 0.440 |
|  | Temporal | 0.197 | 0.019 | 0.665 | 0.716 |
|  | Parietal | 0.173 | 0.017 | 0.685 | 0.716 |
|  | Occipital | 0.000 | 0.000 | 0.990 | 0.990 |
| **Supplementary Table S34** PERMANOVA results comparing False Awakening and REM sleep across frequency bands in the central region for Subject 3. FDR-adjusted p-values (Benjamini-Hochberg method) are reported. | | | | | |

| **Supplementary Table S35** Spectral power differences between False Awakening and Wakefulness for Subject 3. | | | | | |
| --- | --- | --- | --- | --- | --- |
| Frequency Band | Brain Region | F-value | R² | p-value | Adjusted p-value |
| Delta | Frontal | 6.217 | 0.383 | 0.022 | 0.030 |
|  | Central | 12.499 | 0.556 | 0.008 | 0.017 |
|  | Temporal | 10.113 | 0.503 | 0.013 | 0.022 |
|  | Parietal | 14.764 | 0.596 | 0.009 | 0.017 |
|  | Occipital | 11.788 | 0.541 | 0.011 | 0.019 |
| Theta | Frontal | 25.702 | 0.720 | 0.003 | 0.015 |
|  | Central | 24.133 | 0.707 | 0.005 | 0.015 |
|  | Temporal | 22.355 | 0.691 | 0.004 | 0.015 |
|  | Parietal | 15.653 | 0.610 | 0.005 | 0.015 |
|  | Occipital | 14.983 | 0.600 | 0.004 | 0.015 |
| Alpha | Frontal | 24.204 | 0.708 | 0.002 | 0.015 |
|  | Central | 25.804 | 0.721 | 0.004 | 0.015 |
|  | Temporal | 21.173 | 0.679 | 0.007 | 0.017 |
|  | Parietal | 12.351 | 0.553 | 0.013 | 0.021 |
|  | Occipital | 11.866 | 0.543 | 0.010 | 0.020 |
| Beta | Frontal | 1.303 | 0.115 | 0.283 | 0.328 |
|  | Central | 2.778 | 0.217 | 0.130 | 0.171 |
|  | Temporal | 0.637 | 0.060 | 0.482 | 0.492 |
|  | Parietal | 2.452 | 0.197 | 0.164 | 0.196 |
|  | Occipital | 0.877 | 0.081 | 0.378 | 0.407 |
| Low-Gamma | Frontal | 11.707 | 0.539 | 0.010 | 0.017 |
|  | Central | 27.163 | 0.731 | 0.003 | 0.015 |
|  | Temporal | 21.294 | 0.680 | 0.003 | 0.015 |
|  | Parietal | 1.052 | 0.095 | 0.357 | 0.394 |
|  | Occipital | 0.028 | 0.003 | 0.874 | 0.873 |
| **Supplementary Table S35** PERMANOVA results comparing False Awakening and Wakefulness across frequency bands in the central region for Subject 3. FDR-adjusted p-values (Benjamini-Hochberg method) are reported. | | | | | |

**Subject 6**

| **Supplementary Table S36** Post hoc PERMANOVA results for Subject 6. | | | | | | |
| --- | --- | --- | --- | --- | --- | --- |
| Comparison | Df | Sum of Squares | F-value | R² | p-value | Adjusted p-value |
| False Awakening vs REM sleep | 1 | 0.182 | 5.718 | 0.364 | 0.038 | 0.227 |
| False Awakening vs S1 | 1 | 0.257 | 9.575 | 0.489 | 0.006 | 0.036 |
| False Awakening vs Wakefulness | 1 | 0.126 | 3.069 | 0.235 | 0.036 | 0.217 |
| REM sleep vs Wakefulness | 1 | 0.257 | 10.051 | 0.501 | 0.003 | 0.015 |
| S1 vs REM sleep | 1 | 0.039 | 3.434 | 0.256 | 0.023 | 0.137 |
| S1 vs Wakefulness | 1 | 0.293 | 14.229 | 0.587 | 0.003 | 0.016 |
| **Supplementary Table S36** Post hoc PERMANOVA results comparing False Awakening and control conditions (REM sleep, S1, and Wakefulness) for Subject 6. P-values were Bonferroni-adjusted for multiple comparisons.. | | | | | | |

| **Supplementary Table S37** Spectral power differences between False Awakening and S1 for Subject 6. | | | | | |
| --- | --- | --- | --- | --- | --- |
| Frequency Band | Brain Region | F-value | R² | p-value | Adjusted p-value |
| Delta | Frontal | 15.958 | 0.615 | 0.009 | 0.018 |
|  | Central | 0.425 | 0.041 | 0.527 | 0.517 |
|  | Temporal | 0.746 | 0.069 | 0.417 | 0.434 |
|  | Parietal | 0.930 | 0.085 | 0.351 | 0.374 |
|  | Occipital | 4.715 | 0.320 | 0.064 | 0.076 |
| Theta | Frontal | 4.393 | 0.305 | 0.069 | 0.076 |
|  | Central | 21.514 | 0.683 | 0.002 | 0.007 |
|  | Temporal | 27.527 | 0.734 | 0.002 | 0.007 |
|  | Parietal | 53.226 | 0.842 | 0.002 | 0.007 |
|  | Occipital | 17.402 | 0.635 | 0.002 | 0.007 |
| Alpha | Frontal | 8.574 | 0.462 | 0.027 | 0.034 |
|  | Central | 7.078 | 0.414 | 0.042 | 0.055 |
|  | Temporal | 8.617 | 0.463 | 0.016 | 0.024 |
|  | Parietal | 9.720 | 0.493 | 0.018 | 0.028 |
|  | Occipital | 13.095 | 0.567 | 0.005 | 0.009 |
| Beta | Frontal | 5.017 | 0.334 | 0.054 | 0.072 |
|  | Central | 16.085 | 0.617 | 0.003 | 0.007 |
|  | Temporal | 32.340 | 0.764 | 0.002 | 0.007 |
|  | Parietal | 13.597 | 0.576 | 0.005 | 0.009 |
|  | Occipital | 9.117 | 0.477 | 0.007 | 0.013 |
| Low-Gamma | Frontal | 12.450 | 0.555 | 0.011 | 0.018 |
|  | Central | 76.299 | 0.884 | 0.002 | 0.007 |
|  | Temporal | 59.285 | 0.856 | 0.003 | 0.007 |
|  | Parietal | 52.153 | 0.839 | 0.003 | 0.007 |
|  | Occipital | 42.541 | 0.810 | 0.003 | 0.007 |
| **Supplementary Table S37** PERMANOVA results comparing False Awakening and S1 across frequency bands in the central region for Subject 6. FDR-adjusted p-values (Benjamini-Hochberg method) are reported. | | | | | |

**Subject 7**

| **Supplementary Table S38** Post hoc PERMANOVA results for Subject 7. | | | | | | |
| --- | --- | --- | --- | --- | --- | --- |
| Comparison | Df | Sum of Squares | F-value | R² | p-value | Adjusted p-value |
| False Awakening vs REM sleep | 1 | 0.084 | 3.282 | 0.247 | 0.057 | 0.344 |
| S1 vs False Awakening | 1 | 0.127 | 3.036 | 0.233 | 0.048 | 0.286 |
| False Awakening vs Wakefulness | 1 | 0.375 | 12.442 | 0.554 | 0.003 | 0.020 |
| REM sleep vs Wakefulness | 1 | 0.740 | 51.465 | 0.837 | 0.003 | 0.015 |
| S1 vs REM sleep | 1 | 0.115 | 4.421 | 0.307 | 0.017 | 0.099 |
| S1 vs Wakefulness | 1 | 0.507 | 16.510 | 0.623 | 0.002 | 0.011 |
| **Supplementary Table S38.** Post hoc PERMANOVA results comparing False Awakening and baseline conditions (REM sleep, S1, and Wakefulness) for Subject 7. P-values were Bonferroni-adjusted for multiple comparisons. | | | | | | |

| **Supplementary Table S39** Spectral power differences between False Awakening and Wakefulness for Subject 7. | | | | | |
| --- | --- | --- | --- | --- | --- |
| Frequency Band | Brain Region | F-value | R² | p-value | Adjusted p-value |
| Delta | Central | 10.738 | 0.518 | 0.002 | 0.004 |
| Theta | Central | 18.886 | 0.654 | 0.004 | 0.006 |
| Alpha | Central | 7.443 | 0.427 | 0.022 | 0.020 |
| Beta | Central | 11.599 | 0.537 | 0.002 | 0.004 |
| Low-Gamma | Central | 10.701 | 0.517 | 0.002 | 0.004 |
| **Supplementary Table S39** PERMANOVA results comparing False Awakening and Wakefulness across frequency bands in the central region for Subject 7. FDR-adjusted p-values (Benjamini-Hochberg method) are reported. | | | | | |

**Subjective Descriptions of Non-Ordinary States of Consciousness and Representative EOG/EEG/EMG Segments from Canonical Sleep Stages**

| **Supplementary Table S40. First-person reports illustrating each type of non-ordinary state of consciousness (NOSC) during sleep.** | | |
| --- | --- | --- |
| Subject | Type of NOSC | Report |
| 1 | Lucid Dream | I was in a room with three women who were watching a computer screen. They told me I had to move my eyes, **that’s when I realized I was dreaming** and remembered I had to leave the eye mark. I started moving my eyes, and they congratulated me. I remember the happiness of being aware in the dream and being able to leave the mark. |
| 2 | Lucid Dream | I was in a place with many people and started talking to someone. I don’t remember what we talked about, but that’s when **I realized I was dreaming. I automatically left the eye mark.** I continued in the dream, but I don’t remember much else. |
| 3 | Lucid Dream | I remember being in a sort of park, a cement courtyard. **At one point, when I had lucidity, I started to leave the mark.** I counted them; I can't remember well now, but I think I left three marks, going quite slowly. There was a girl, I think she was a former classmate from biology in college. I was leaving the mark and wanted to return to the laboratory. It was as if it were a park next to the laboratory. I went back to the laboratory, and that girl was asking me why I was returning or what I was doing with the mark or something like that. So I told her to wait and let me finish the mark. I went into a kind of laboratory, but it was something else, some building I recognized, but I don't remember where. I don't know if it was a mix of the fine arts faculty in L.P. or some physics or chemistry laboratory where I used to study. It was a mix of things from laboratories I knew. I was trying to look there, and I got lost. I don't remember more. |
|  | OBE_1_ | **I left my body,** and I think there was another person sleeping too, a person with a pom-pom hat (haha). **I immediately left the mark several times**. I went out through the door, passing through it. I walked down the hallway where there were boxes, like phone booths. I didn’t want to look there for fear of seeing something. I walked through that area, and as I passed them, I encountered students taking a class, many of them. A girl looked at me. She had gray hair, glasses, and was skinny. She seemed to greet me or something. I approached them. **I left the mark again several times.** Someone said something to me about the girl, like they were teasing me or something. I approached, and the girl, following that teasing, hugged me. Then I woke up |
|  | OBE_2_ | **I left my body** and walked with great difficulty. Behind the door, there was something else, researchers too, students, etc., but it was much more crowded and social. I could hear my breathing a lot, like snoring. I didn’t make the eye signal. It was very hard for me to move, and I asked for clarity, but it was very difficult to get out of the room. I think there was a kind of sideboard on the right side of the room, next to the bedside table, on the wall adjacent to the other room, from my point of view while lying down. I tried to read something, but I don’t remember; there were notes. The computers looked out onto a kind of glass wall, like a recording studio, the “fishbowl,” as they call it, but on the other side, it just felt like there were people. It didn’t feel like they were watching people sleep or anything. |
|  | False Awakening | **I thought I was awake**, hearing people walking outside the room, taking other researchers on a tour. But something happened, and they started arguing, to the point where they began to "break" the lab. There were loud screams, etc. And they tugged at the cables of the device that connects the electrodes. I was being pulled toward the door. In other words, instead of being pulled back toward the wall (where the cables come from), I was being pulled toward the door. And that's when I woke up, clearly, that was a dream. But the feeling of being pulled was very strong. |
| 4 | Sleep Paralysis | At first, I was thinking that I couldn't fall asleep, and then everything went black. I saw my dad on my left side, crouched down, fixing something. There was a hole in the floor, and he was putting tools in there. The sounds of the metal tools hitting the hole seemed very real to me. **That’s when I left a mark and waited a little bit before leaving another one, just in case**. Then I hurried to wake up because I saw a black shadow, like a floating cloud, chasing me, and it felt very real too. **I was in sleep paralysis the whole time.** |
| 5 | Sleep Paralysis | I turn around, look at the right wall, and try to fall asleep. Before that, I realize the atmosphere feels warm, that’s how I usually describe **the onset of sleep paralysis**. The air feels heavy, like stale air; it's unpleasant. I lie down, apparently fall asleep quickly, **and then the paralysis begins**. I hear a noise, like a terrible horror. I feel as if everything is filled with green gases, green colors, like moss green, as if I’m in a polluted atmosphere. **I felt a presence.** It wasn’t a monster or something I could see, but I could feel it, and I knew it could do something bad to me. That feeling was awful. |
| 6 | False Awakening | I dreamed that they came to wake me up and take off the electrodes (it was very real; **I thought I was awake**). Nerea came in, thanked me for participating, and said she wanted to show me what had been recorded and tell me what she had seen. Meanwhile, I told her about my dreams (several normal dreams, but I told her about a funny work-related one). She took off all the electrodes, and I was ready to leave, but before that, she told me to look at a monitor inside the room and showed me some “graphs.” Then she told me to keep talking about A. (a co-worker I had dreamed about), and that’s when I realized I had never told her A.’s name, and that there was no monitor or computer in the real room, and that Nerea’s face was starting to change. **That’s when I realized it was a dream and “woke up” again**. **In that other “awakening,” I also dreamed that they were coming to take everything off. The room was the same as the real one.** As we went down the hallway, just after leaving the room, I saw a man with a box, which caught my attention because it seemed somewhat out of context and didn’t seem like he belonged to the laboratory. At the same time, I began to feel my body in the bed, in the side position, the blankets, and everything exactly as I was sleeping. I also felt the tape and the electrode on my chin, and that’s when I realized I was still asleep and that it was another false awakening. And then I woke up for real, and I knew it was real because I was in the same position I had perceived during the dream, and also because I usually feel like it’s my real body when I feel a warmth or “electricity” (I don’t know how to describe it) that runs through my body, something different from what I feel in the body I have when dreaming. |
| 7 | False Awakening | **I woke up in the lab bed** and started taking off the electrodes. While I was sitting there doing that, we started talking about my nap. **That’s when I realized I was dreaming, that it was a false awakening.** I started moving my eyes very fast to leave the mark, and then I woke up. |
| **Supplementary Table S40.** One representative excerpt is provided for each reported experience: lucid dreaming (LD), sleep paralysis (SP), out-of-body experience (OBE), and false awakening (FA). These reports were used to classify each episode based on phenomenological criteria defined in the study. Key phrases that were central to the classification of the episode type are highlighted in bold. | | |

**
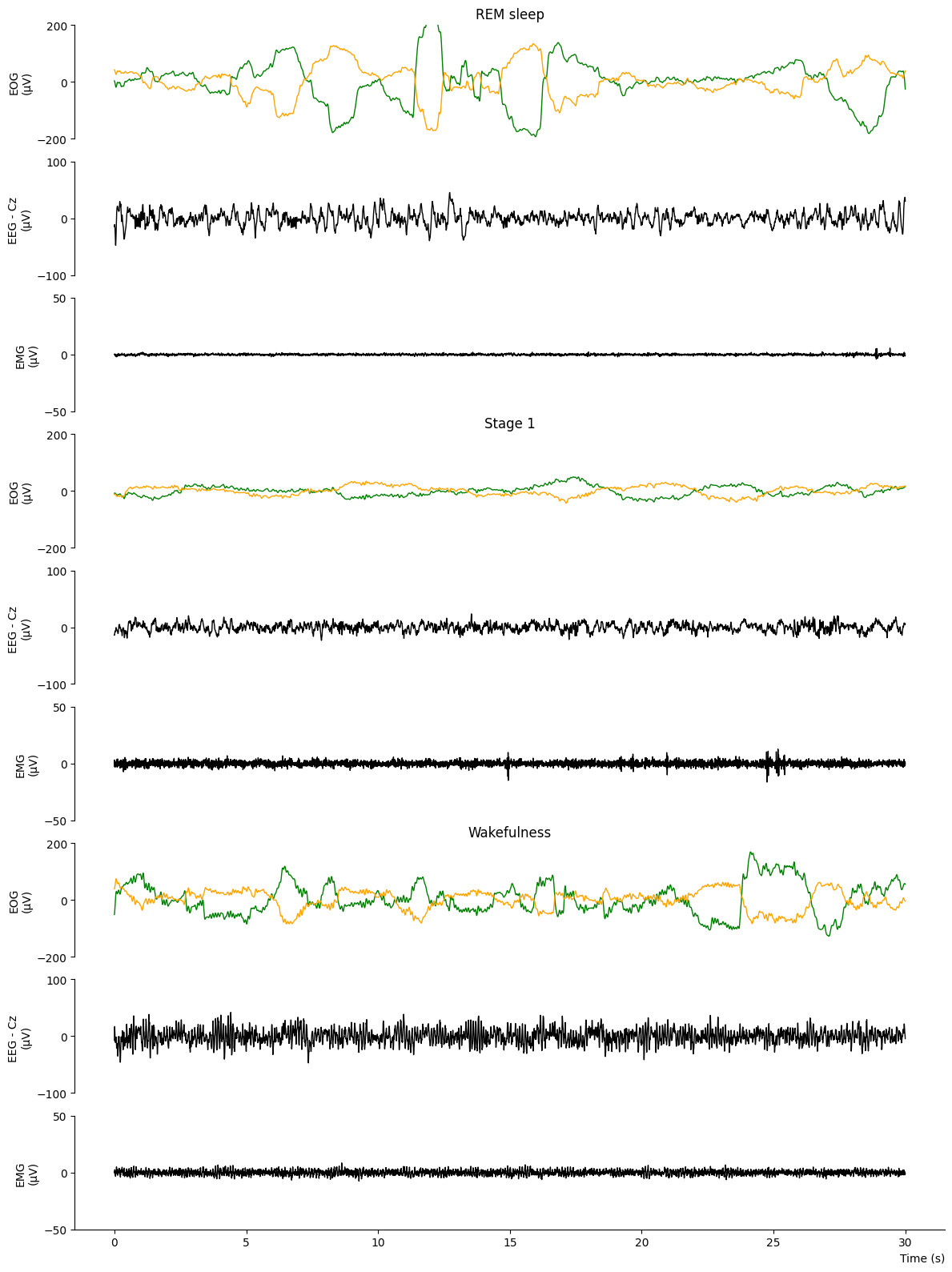
Supplementary Figure S11. Representative 30-second EOG/EEG/EMG segment from canonical sleep stages in Subject 2.** This real recording was selected to illustrate the characteristic patterns of REM sleep, Stage 1 and Wakefulness. EEG (channel Cz), EMG, and EOG traces are shown.
